# Stability of Ternary Drug–Drug–Drug Coamorphous Systems Obtained Through Mechanochemistry

**DOI:** 10.3390/pharmaceutics17010092

**Published:** 2025-01-12

**Authors:** Ilenia D’Abbrunzo, Elisabetta Venier, Francesca Selmin, Irena Škorić, Enrico Bernardo, Giuseppe Procida, Beatrice Perissutti

**Affiliations:** 1 Department of Chemical and Pharmaceutical Sciences, University of Trieste, Piazzale Europa 1, 34127 Trieste, Italyelisabetta.venier@studenti.units.it (E.V.); gprocida@units.it (G.P.); 2 Department of Pharmaceutical Sciences, University of Milan, Via G. Colombo, 71, 20133 Milan, Italy; francesca.selmin@unimi.it; 3 Department of Organic Chemistry, Faculty of Chemical Engineering and Technology, University of Zagreb, Marulićev trg 19, 10000 Zagreb, Croatia; iskoric@fkit.unizg.hr; 4 Department of Industrial Engineering, University of Padova, Via F. Marzolo 9, 35131 Padova, Italy; enrico.bernardo@unipd.it

**Keywords:** ternary coamorphous, binary coamorphous, drug-to-drug-to-drug coamorphous, glass transition, physical stability, storage temperature

## Abstract

**Background/Objectives:** This study investigates the preparation of coamorphous systems composed entirely of active pharmaceutical ingredients (APIs), namely praziquantel, niclosamide, and mebendazole. The objective was to formulate and characterize binary and ternary coamorphous systems to evaluate their structural, thermal, and stability properties. **Methods:** Ten different mixtures (binary and ternary) were designed through a mixture design approach and prepared using a sustainable, one-step neat grinding process in a lab-scale vibrational mill. The systems were prepared reproducibly within 4 h across the entire experimental domain. Structural characterization was performed using PXRD and FTIR to confirm the absence of crystalline domains and the presence of molecular interactions. The glass transition temperature (T_g_) was theoretically calculated using the Gordon–Taylor equation for three-component systems and determined experimentally via DSC. Stability studies were conducted on seven systems under different storage conditions (−30 °C, 5 °C, 25 °C, and 40 °C) for six months. **Results:** PXRD analysis confirmed the formation of coamorphous systems with no crystalline phases. DSC revealed a single T_g_ for most systems, indicating homogeneity. Stability studies demonstrated that five out of seven systems adhered to the “T_g_—50 °C” stability rule, remaining physically stable over six months. Recrystallization studies indicated diverse pathways: some systems reverted to their original crystalline phases, while others formed new entities such as cocrystals. **Conclusions:** This study highlights the feasibility of coamorphous systems composed of multiple APIs using a simple, solvent-free grinding approach. The findings underscore the importance of molecular interactions in determining stability and recrystallization behavior, offering insights for designing robust coamorphous formulations.

## 1. Introduction

Coamorphous systems offer a promising approach in pharmaceutical formulations, presenting distinct advantages over both crystalline and traditional amorphous forms. Among these benefits are the enhanced solubility and bioavailability of poorly water-soluble drugs. By stabilizing drugs in the amorphous state with suitable coformers, coamorphous systems help overcome the solubility challenges that typically affect crystalline drugs, leading to faster dissolution rates that are crucial for ensuring consistent and rapid drug absorption in vivo [1,2]. Additionally, when properly formulated, coamorphous systems exhibit superior physical stability compared to individual amorphous forms, which are often unstable and prone to recrystallization when subjected to storage or environmental stresses [3]. The intimate molecular-level mixing of drugs with coformers in coamorphous systems reduces the molecular mobility and significantly lowers the risk of phase separation or crystallization [4,5,6,7,8,9].

Traditionally, coamorphous systems consist of two low-molecular-weight coformers (e.g., drugs, amino acids, dicarboxylic acids, flavonoids, sugars, etc., [8,10,11]) and exhibit a single glass transition temperature (T_g_). A 1–1 molar ratio is quite common in coamorphous systems [7,12], resulting thus in higher drug-loading compared to classical amorphous solid dispersions. Recent advancements are increasingly focusing on the design of three-component coamorphous systems, which, despite being more complex and challenging to formulate due to the need for their optimized molecular compatibility, offer even greater physical stability. The inclusion of a third component could promote stronger molecular interactions, such as hydrogen bonding and van der Waals forces, which further disrupt potential recrystallization pathways, resulting in the superior stabilization of the amorphous state compared to two-component systems [7,13,14].

In this context, where multiple active pharmaceutical ingredients (APIs) are administered simultaneously, drug–drug coamorphous systems offer a valuable platform. These systems can stabilize and enhance the solubility of multiple drugs within a single formulation, facilitating the development of therapies that are not only more effective, but also more convenient for patients. This approach may avoid the complexities and potential drug–drug interactions that often arise with traditional crystalline mixtures or cocrystals [15].

Moreover, coamorphous systems provide flexibility in tailoring drug release profiles to meet specific therapeutic needs. By adjusting the composition of coformers and drug ratios, researchers can fine-tune the release kinetics of the drugs within coamorphous formulations. This capability is especially useful for controlled or sustained drug delivery, where precise release profiles are essential for optimizing therapeutic efficacy while minimizing side effects [16]. Additionally, coamorphous systems often exhibit good processability and scalability, making them suitable for large-scale manufacturing. Unlike nanoparticle or crystalline formulations that may require specialized manufacturing techniques, coamorphous systems can be produced using conventional pharmaceutical processes, such as spray-drying or co-milling, which ensure cost-effective production and broader accessibility [17].

Beyond their functional advantages, the novel compositions and improved properties of coamorphous systems provide opportunities for patent protection and market differentiation. Pharmaceutical companies can capitalize on the unique benefits of coamorphous formulations to extend the patent life, secure market exclusivity, and set their products apart from competitors offering conventional dosage forms.

**Figure 1:**
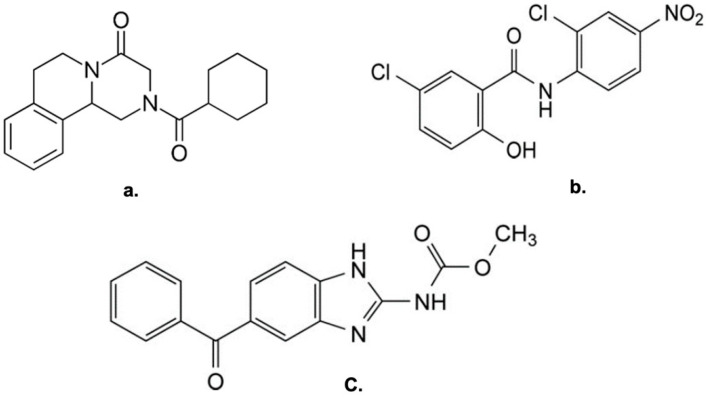
(**a**) Praziquantel, (**b**) niclosamide, and (**c**) mebendazole molecular structures.

Given this context, the objective of this experimental work was to investigate the feasibility of developing drug–drug coamorphous systems—both two-component and three-component—by combining three anthelmintic drugs, planning the trials by means of an experimental design for mixtures: praziquantel (PZQ), niclosamide (NCM), and mebendazole (MBZ) (Figure 1). Specifically, based on the recent development of dual-drug coamorphous systems through mechanochemistry [10,18], this study aimed to determine whether PZQ–NCM–MBZ mixtures could give origin to homogenous coamorphous systems, and whether these systems could be produced through neat grinding (NG), without the need for solvents [8,19,20]. The research focuses on assessing whether such systems could stabilize each drug in its amorphous state, enhancing their stability during storage. By creating coamorphous mixtures, it is assumed that the combination of these drugs will promote molecular-level interactions, such as hydrogen bonding, which could prevent recrystallization and enhance individual amorphous stability. This study compared two-component systems, where one drug acts as a coformer for the other, to three-component systems, where the third drug—if properly selected—is expected to further improve stability through an added intermolecular complexity.

The selection of these APIs, all characterized by anthelmintic activity—which also justifies their use in polytherapy (e.g., PZQ and MBZ [21])—is due to their poor aqueous solubility and low bioavailability [22]. All three APIs have numerous crystalline solid forms indexed in the Cambridge Structural Database (CSD) [23], confirming their ability to interact in the solid state with various coformers. Furthermore, they all have demonstrated the ability to form coamorphous systems [11] or amorphous solid dispersions [24,25]. Additionally, the literature reports several examples of the three drugs used in mechanochemical experiments [26,27].

By enhancing the amorphous stability and improving the T_g_s values of these drugs, this approach has the potential to greatly improve their therapeutic efficacy, addressing the persistent challenges related to drug solubility and delivery.

## 2. Materials and Methods

### 2.1. Materials

PZQ, (RS)-2-(Cyclohexylcarbonyl)-1,2,3,6,7,11b-hexahydro-4-H-pyrazino[2,1-a]-isoquinolin-4-one], of Ph. Eur. grade was kindly donated by Fatro S.p.a. (Bologna, Italy), while NCM (5-chloro-N-(2-chloro-4-nitrophenyl)-2-hydroxybenzamide) (purity of 98–101%) and MBZ (methyl N-(6-benzoyl-1H-benzimidazol-2-yl)carbamate) (purity ≥ 98%) were purchased from Sigma-Aldrich (St. Louis, MO, USA). All the actives were used without further purification before grinding.

### 2.2. Methods

#### 2.2.1. Samples Preparation

##### Mixture Composition and Experimental Design

The composition of the mixtures was formulated using a mixture design approach [28], implemented via NEMRODW software (Ed. 2017) [29].

The mixtures subjected to grinding consisted of three powdered components: praziquantel (PZQ, X1), mebendazole (MBZ, X2), and niclosamide (NCM, X3). The limits of each component in the mixtures were defined during the preliminary trials obtaining the following values: 0 ≤ X ≤ 0.5 (expressed as a molar ratio in the mixture). In fact, the mixture design approach was used exclusively for exploratory analysis.

**Figure 2:**
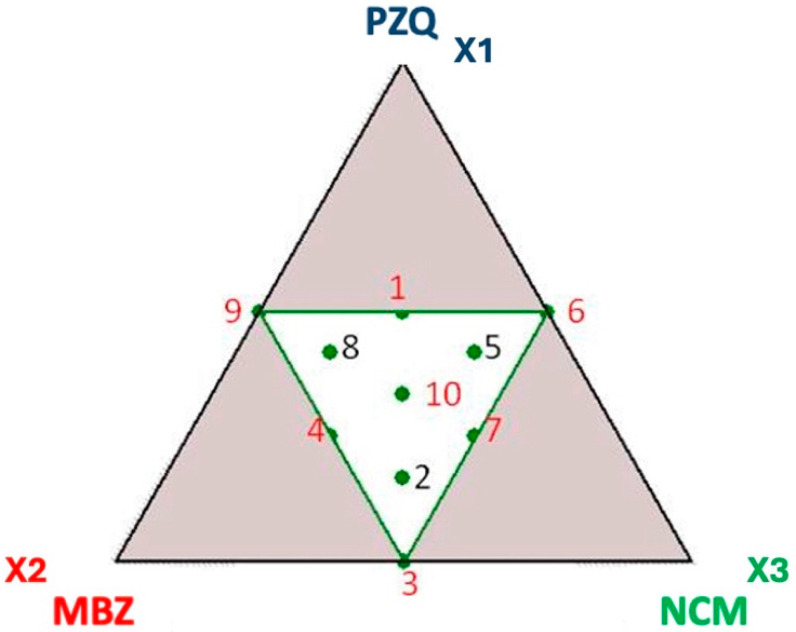
Ternary diagram representing the experimental design space. X1, X2, and X3 represent the molar ratios of the drugs.

The experimental matrix is detailed in Table 1, and the ternary diagram representing the experimental design space is shown in Figure 2.

##### Neat Grinding (NG) Experiments

Milling was carried out using a Retsch MM400 vibrational mill (Retsch, Germany) equipped with two 25 mL stainless steel jars, each containing a 10 mm Ø stainless steel bead. The milling frequency was consistently set at 25 Hz for all experiments, with the void volume in the jars kept constant by maintaining a total powder mass of 400 mg per 25 mL jar. The milling duration was 4 h.

To ensure reproducibility, each mixture was milled in at least four replicates. Pure crystalline drugs (MBZ and NCM) (400 mg per drug) were also milled individually under the same conditions. PZQ was not milled alone, as its behavior under these conditions had already been well-documented in previous studies [30,31].

##### Physical Mixtures’ Preparation

Physical mixtures were prepared by manually mixing the active ingredients in an agate mortar for a standardized time of 3 min. Both binary and ternary mixtures were prepared, with a total mass of 400 mg for each batch. The molar ratios of the APIs were varied according to the experimental mixture design (Table 1).

#### 2.2.2. Samples Characterization

##### Powder X-Ray Diffraction (PXRD)

PXRD analysis was carried out by a Bruker D2 Phaser benchtop diffractometer (Bruker, Manheim, Germany) using the Bragg–Brentano geometry, using Cu-Kα radiation (λ = 1.5418 Å) with a 300 W low-power X-ray generator (30 kV at 10 mA). All the measurements were conducted in a 2θ range of 3–40° with a step size of 0.02° and a scan speed of 0.6°/s. Each sample was prepared by gently pressing approximately 200 mg of ground product into the cavity of a steel sample holder equipped with a cylindrical polyvinylidene fluoride (PVDF) reducer.

To minimize the amount of powder used and the enable analysis of the same sample throughout the entire period of study, a special zero-background sample holder was employed for PXRD analyses of the coamorphous system during stability studies over the 6-month period.

PXRD patterns were recorded for the starting materials, physical mixtures, all milled samples, and a selection of aged coamorphous samples.

##### Differential Scanning Calorimetry (DSC)

Selected samples, weighing 2–4 mg, were introduced into an aluminum sealed and pierced 40 μL crucible and analyzed by a Mettler Toledo DSC 3 Star System (Milan, Italy) using STARe software version 16.40 for the data analysis. The heating program employed a temperature ramp of 10 °C/min, from 30 °C to 350 °C, under a nitrogen (N_2_) flow rate of 50 mL/min.

The following samples were analyzed: pure APIs, physical mixtures, coamorphous samples produced via NG, and a selection of aged coamorphous samples.

DSC was mainly applied to experimentally evaluate the T_g_s of the ten coamorphous samples and the T_g_ temperature was expressed as an inflection point in the thermal curve.

To obtain MBZ T_g_, DSC heating/cooling/heating cycles were applied. These cycles were performed under a nitrogen (N_2_) flow of 50 mL/min, following this protocol: 1. Heating from 30 °C to 335 °C at a rate of 10 °C/min. 2. Isotherm at 335 °C for 5 min. 3. Cooling from 335 °C to −30 °C at a rate of 50 °C/min. 4. Isotherm at −30 °C for 5 min. 5. Reheating from −30 °C to 330 °C at a rate of 40 °C/min.

In certain cases, such as with NCM, where the T_g_ could not be detected using conventional DSC analysis, modulated DSC (MTDSC) was performed by using a DSC1 Star^e^ System (Mettler Toledo, CH, Greifensee, Schweiz) equipped with a refrigerated cooling system (RCS). Samples of about 10 mg were quantitatively transferred to pin-holed aluminum pans, sealed, weighed, and subjected to heating cycles from 10 to 350 °C (1 K/min) for a period of 90 s and an amplitude 0.5 °C. The DSC cell and RCS were purged with dry nitrogen at 80 and 120 mL/min, respectively. The system was calibrated using an indium standard. All data were treated with Star^e^ System software (V16.40) (Mettler Toledo, CH, Greifensee, Switzerland).

##### True Density Determinations

The true density of the three pure powdered APIs was measured using a helium pycnometer (Ultrapyc 5000, Anton Paar GmbH, Graz, Austria). The device is based on gas pressure determinations in connected, sealed chambers of a specified volume. At first, a chamber is filled with powders and gas; the latter is later allowed to flow in a second chamber, free of powders. The difference in the helium pressure in the two chambers is used to calculate the volume occupied by the powders, using Boyle’s law [32]. The density is finally determined by the ratio between the mass of powders and the calculated volume. The accuracy of the measurement relies on the penetration of inert gas in any void between particles. The density values of crystalline materials were used as crude density values for the amorphous components and serve to calculate the constant, K (Equation (1)):

(1) $$K=\frac{{Tg}_{1}\times \rho_{1}}{{Tg}_{2}\times \rho_{2}}$$

K is a fundamental constant of the Gordon–Taylor (G-T) equation (Equation (2)), which considers the weight fractions and T_g_ values of the individual component, as well as the true density values of their powders, to calculate a theoretical T_g_ for a coamorphous system [33,34]:

(2) $${Tg}_{mix}=\frac{\left[\left(w_{1}{Tg}_{1}\right)+\left(K_{2}w_{2}{Tg}_{2}\right)+\left(K_{3}w_{3}{Tg}_{3}\right)\right]}{\left[{(w}_{1}+\left(K_{2}w_{2}\right)+\left(K_{3}w_{3}\right)\right]}$$

where pedix 1, 2, and 3 refer to PZQ, NCM, and MBZ, respectively.

The T_g_ values included in the equation for the individual components are the experimentally determined values, as measured and described in Section Differential Scanning Calorimetry (DSC) 2.

##### Fourier Transform Infrared Spectroscopy (FT-IR)

Powdered samples were analyzed with a Shimadzu IRAffinity-1S FT-IR instrument (Kyoto, Japan) in a range of 400–4000 cm^−1^ with a resolution of 4 cm^−1^ and 20 scans.

Suitable discs for analysis were prepared by gently grinding in an agate mortar 200 mg of anhydrous KBr (99% infrared grade) with 1% *w*/*w* of analyte and then pressing the mixture with a hydraulic press (PerkinElmer, Norwalk, CT, USA), applying a pressure of 10 Ton for 3 min.

The data interpretation and comparisons were performed using SpectraGryph 1.2 software.

##### Scanning Electron Microscopy (SEM)

Images of some coamorphous were collected through SEM. The powdered sample was placed on an aluminum stub covered with carbon double-sided tape and sputter-coated with gold using a Sputter Coater K550X (Emitech, Quorum Technologies Ltd., Lewes, UK), before being analyzed by a scanning electron microscope (Quanta 250 SEM, FEI, Hillsboro, OR, USA) with the secondary electron detector. The working distance was set at 10 mm to obtain the appropriate magnifications, and the acceleration voltage was set at 5 kV.

##### Stability Studies According to “T_g_—50 °C” Rule

Selected samples (i.e., exp. N° 1, 3, 4, 6, 7, 9, and 10) were stored at four different temperatures (−30 °C, 5 °C, 25 °C, and 40 °C) for 6 months to assess their physical stability according to the “T_g_—50 °C” rule under various temperature conditions. To protect the samples from atmospheric humidity, they were stored in sealed vials placed inside sealed plastic bags. Samples were withdrawn during the first month on a weekly basis and then on a biweekly basis and tested to investigate the possible recrystallization using PXRD. Samples stored for 6 months under the most severe conditions (i.e., −30 °C and 40 °C) were also evaluated for drug recovery (see section below).

Additionally, it is well-established in the literature that amorphous systems are prone to hygroscopicity [35,36]. Therefore, to gather preliminary data on the effects of the relative humidity (RH) on these coamorphous systems, selected samples were further analyzed using the dynamic vapor sorption (DVS) technique. Specifically, samples from exp. N° 3, 6, 9, and 10 were placed in crucibles and subjected to a gradual RH increase from 0% to 90% at a constant temperature of 25 °C. This allowed for the evaluation of mass changes, providing insight into surface moisture absorption and, consequently, powder hygroscopicity.

##### Drug Recovery

To check possible degradation of coground samples after preparation, ^1^H-NMR spectra were recorded on a Bruker Avance 600 spectrometer equipped with a 14 T superconducting magnet and two 5 mm probes. Analyzed samples were dissolved in chloroform deuterated (CDCl_3_), using tetramethyl silane (TMS) as a reference. Measurements were conducted at 300 K using a simple pulse–acquire sequence. Drug recovery analyses were performed on the same seven samples (i.e., exp. N° 1, 3, 4, 6, 7, 9, and 10) used for the physical stability study, specifically those stored in the freezer and those subjected to stressed conditions at 40 °C.

## 3. Results and Discussion

The first step of this experimental work involved the planning of an experimental matrix using the NEMROWD software to design binary and ternary mixtures of the three APIs (PZQ, NCM and MBZ). The mixture design approach was used in our study only for carrying out an exploratory analysis inside the design space (i.e., to rationalize mixture compositions, instead of a random selection approach of compositions). This is therefore not a DoE aimed at defining a predictive/descriptive model for the experimental responses. We used intentionally a simple experimental plan that included binary mixtures (placed at the vertices of the outer triangle) and ternary mixtures (within the inner triangle), with specific limits to deliberately exclude experimental points with a single component (as these would not be mixtures). Additionally, we did not want components in very distant proportions (i.e., with one component highly diluted), in order to promote interactions between the components themselves. Since the components are small molecules and not polymers, as is often the case with co-amorphous systems, we expect the interactions to be enhanced in a 1:1 ratio.

Once these constraints were established, ten different mixtures of the three APIs were obtained (refer to Table 1 for the N° of the experiment and compositions). These ten mixtures were then subjected to NG for 4 h at a frequency of 25 Hz (in batches, each weighing 400 mg).

As a control, physical mixtures with the same compositions as those in the experimental matrix were also prepared. This allowed for a detailed comparison between the coground samples and their unprocessed counterparts, providing valuable insight into the structural differences between the two.

The solid-state nature of the coground samples and physical mixtures was then assessed through PXRD analysis. Diffractograms of all ten milled mixtures revealed a typical halo pattern characteristic of amorphous compounds (see Figures S1–S10 in the Supporting Information (SI) file) [37], in contrast to the physical mixtures, where the PXRD patterns simply reflected the combined crystalline patterns of the two or three (depending on the mixture composition) starting materials (Figure S11 in the SI file). This comparison clearly indicated that manual physical mixing was insufficient to amorphize the three APIs, whereas NG was essential to achieve complete amorphization in all ten systems.

At this stage, some coground samples (all three binary systems plus one ternary system, i.e., exp. N° 3, 6, 9, and 10) were analyzed using FT-IR spectroscopy to investigate the presence of possible interactions between the components, which would indicate the formation of coamorphous systems rather than mere physical blends of the three amorphous components.

**Figure 3:**
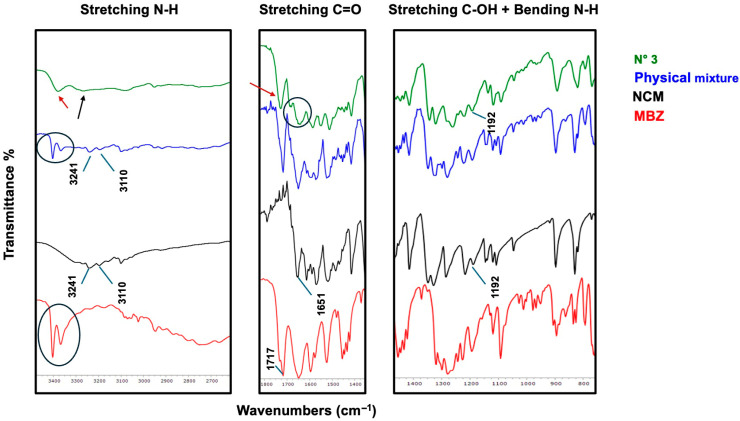
FT-IR spectra of NCM-MBZ 1–1 coamorphous (black) compared with its physical mixture (red) and pure components (blue line represents NCM and light green line MBZ).

Figure 3 shows the FT-IR spectrum of exp. N° 3 (i.e., binary NCM-MBZ 1–1) compared to the corresponding physical mixture and pure components. Starting with MBZ, the N-H amide stretching band at 3404 cm^−1^ [38] is still present in the physical mixture, whereas it disappears in the coamorphous spectrum, indicating their involvement in hydrogen bonding. Similarly, the amide C=O stretching band at 1717 cm^−1^ [38] is no longer detectable in the coamorphous system, suggesting that the carbonyl amide group could also be involved. At 1648 cm^−1^ [38], the benzoyl C=O stretching of MBZ exhibits broadening in the coamorphous spectrum, though the extent of its involvement remains uncertain.

Turning to NCM, the functional group clearly involved in interactions is the N-H, as its stretching bands at 3241 and 3110 cm^−1^ [39], still visible in the physical mixture, completely disappear in the coamorphous spectrum. At 1651 cm^−1^ [40], the C=O stretching band shows broadening in the coamorphous spectrum, though its involvement in hydrogen bonding is less clear. Additionally, the C-OH stretching band at 1192 cm^−1^ [40] appears in both spectra, suggesting no interaction with this group. In conclusion, the N-H amide groups from both components and the MBZ amidic carbonyl appear to be involved in hydrogen bonding.

**Figure 4:**
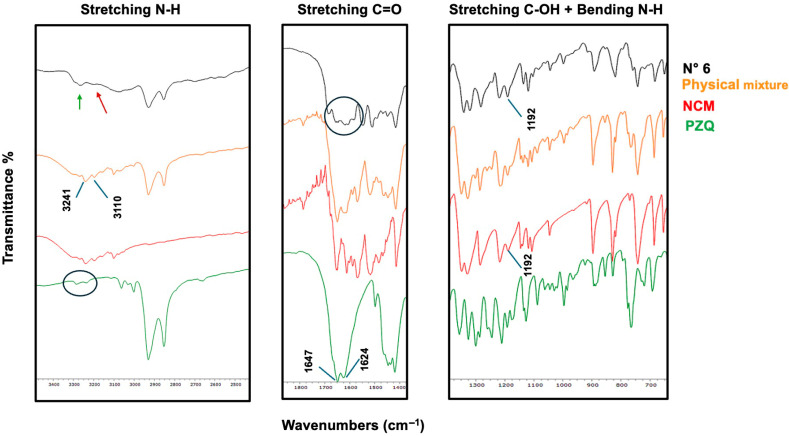
FT-IR spectra of PZQ-NCM 1–1 coamorphous (black) compared with its physical mixture (red) and pure components (blue line represents PZQ and light green line NCM).

Also, the FT-IR of exp. N° 6 (i.e., binary PZQ-NCM 1–1) was useful to elucidate some possible interactions between the two components. The FTIR spectrum of the new coamorphous system exhibits the typical band-broadening characteristic of amorphous materials, differently from the pure crystalline components and the physical mixture. This broadening is particularly evident in the wavenumber region between 3241 and 3110 cm^−1^, where the N-H stretching bands of NCM are located [39], making it difficult to clearly determine whether the donor group is involved in hydrogen bonding. Additionally, the band at 1624 cm^−1^, corresponding to the C=O stretching of PZQ [24,30,41,42], disappears in the coamorphous spectrum, confirming the involvement of the carbonyl group as a hydrogen bond acceptor. Conversely, the C-OH stretching band of NCM at 1192 cm^−1^ [40] is still present in both the physical mixture and the coamorphous spectra, suggesting that this group is not acting as a hydrogen-bond donor. The broadening observed in the coamorphous spectrum makes it difficult to definitively identify all the groups involved in hydrogen bonding. However, a PZQ carbonyl group seems to be implicated as a hydrogen bond acceptor. Figure 4 shows the FT-IR spectra described above.

**Figure 5:**
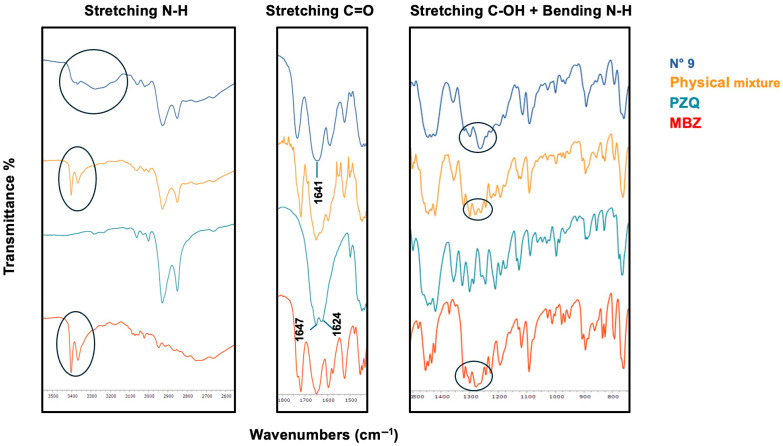
FT-IR spectra of PZQ-MBZ 1–1 coamorphous (blue) compared with its physical mixture (orange) and pure components (light blue line represents PZQ and red line MBZ).

The FT-IR spectra of exp. N° 9 (i.e., binary PZQ-MBZ 1–1) are reported in Figure 5. By comparing the FT-IR spectra of the new coamorphous system, its physical mixture, and the individual components, the presence of solid-state interactions can be hypothesized. In the region related to PZQ carbonyl stretching vibrations, the coamorphous system merges into a single broad and shifted carbonyl band at 1641 cm^−1^, compared to the double peak at 1647 and 1624 cm^−1^ observed for anhydrous PZQ [24,30,41,42]. PZQ bands are still visible in the physical mixture, confirming that no interactions involving these functional groups occur in the simple mixture. Regarding MBZ, the sharp N-H amide stretching band at 3404 cm^−1^ [38], which is still prominent in the physical mixture, is absent in the coamorphous system. This could indicate that the N-H amide group participates in hydrogen bonding. Additionally, the band at 1278 cm^−1^, associated with the C-N stretch of the imidazole [38], shows significant broadening in the coamorphous spectrum, suggesting its involvement in interactions. In contrast, no such broadening is observed in the simple physical mixture.

**Figure 6:**
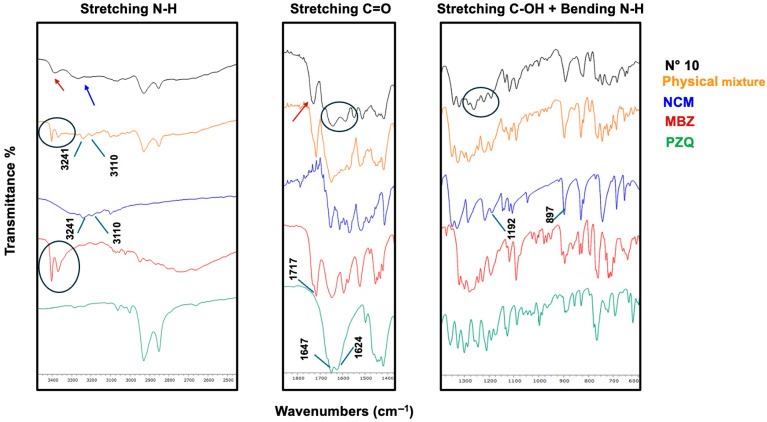
FT-IR spectra of PZQ-NCM-MBZ 1–1-1 coamorphous (black) compared with its physical mixture (red) and pure components (light blue line represents PZQ, blue NCM, and light green line, MBZ).

Figure 6 reports the FT-IR spectra for the ternary PZQ-NCM-MBZ 1–1−1 coamorphous system, the corresponding physical mixture, and the three active ingredients. The FT-IR spectra reveal several informative bands. Considering MBZ, the sharp N-H stretching band at 3404 cm^−1^ [38] completely disappears in the coamorphous spectrum, indicating the involvement of a hydrogen bond. The C-N stretching band at 1278 cm^−1^ [38] shows significant broadening in the ternary system, reflecting its amorphous nature, while the C=O stretching band, originally at 1717 cm^−1^ [38], is absent, confirming the involvement in the interactions. On the contrary, the C=O stretching band at 1648 cm^−1^ [38] remains visible, suggesting that this group is not involved in the interactions. Regarding NCM, the N-H stretching and bending at 3241, 3195 cm^−1^, and 897 cm^−1^ [39], respectively, disappear in the ternary system spectrum, indicating their involvement in hydrogen bonding. The C=O stretching band at 1651 cm^−1^ is significantly broadened but still present, while the C-OH stretching band at 1192 cm^−1^ is broadened and shifted, a behavior also observed in the physical mixture spectrum, making it unclear whether this group participates in hydrogen bonding. As for the C=O groups in PZQ, the carbonyl stretching band at 1624 cm^−1^ disappears in the coamorphous spectrum, indicating its definite involvement in hydrogen bonding. In conclusion, the groups most certainly involved in hydrogen bonding are the amide N-H and carbonyl groups of MBZ, the amide N-H of NCM, and the carbonyl group of PZQ.

Given the complexity of the FT-IR results of coamorphous systems—particularly the ternary ones—and the fact that the starting commercial form of MBZ is itself a mixture of two polymorphs [43] (as also confirmed by DSC and PXRD analyses), the confirmation of the existence of homogeneous coamorphous systems was obtained through the evaluation of the T_g_ of the system, as it is one of the key features that distinguishes amorphous and coamorphous systems from crystalline materials. Particularly regarding coamorphous systems, which are homogeneous multicomponent systems, one would expect to observe a single T_g_ value. Therefore, experimentally determining the T_g_ and confirming a unique value for each of the ten mixtures could provide further evidence that homogeneous coamorphous systems were successfully obtained.

The experimental T_g_ values were primarily determined using conventional DSC analysis. However, in some cases, where it was not possible to detect T_g_ through traditional DSC, MTDSC was employed.

To provide a comparison of the experimental results, theoretical T_g_ values of the (binary or ternary) drug mixtures were also calculated using the Gordon–Taylor (G-T) equation [33,34].

As for the individual APIs, the T_g_ was determined experimentally by DSC (or MTDSC in the PZQ and NCM cases) and compared to theoretical T_g_ values (Figures S12–S14). The experimental values, not in good agreement with the conventional Rule of 2/3 [44], were in satisfactory accordance with the predicted model recently proposed by Alzghoul and coauthors [45], unless for MBZ. The experimental T_g_ values of PZQ and MBZ were found to be consistent with those previously reported in the literature [46,47], while the T_g_ of NCM was experimentally determined for the first time.

Table 2 summarizes the experimental and calculated T_g_ values for the ten mixtures and the three pure APIs.

As highlighted in this table, the experimental T_g_ values of the mixtures obtained via DSC were generally in good agreement with the theoretical values calculated using the G-T equation. The similarity between the experimental T_g_ and the theoretical T_g_ values indicated that there might not be strong molecular interactions between the drugs [11]. In these mixtures, slight positive deviations were observed, generally indicating a slightly higher rigidity of the vitreous state, as also confirmed by FT-IR analyses, which remain to be verified by stability studies [44,48,49].

However, experimental analyses (i.e., DSC and MTDSC) were unable to clearly determine the T_g_ in two out of the thirteen systems investigated. This issue could be attributed to several reasons: the known difficulty in detecting T_g_ in some amorphous formulations prepared by grinding, as these systems occasionally fail to produce a distinct T_g_ signal, possibly due to incomplete amorphization or other physical disruptions introduced during the grinding process [2,50], or simply to the fact that these two systems are not coamorphous systems.

In the remaining eight cases, a single T_g_ value was observed (Figures S15–S22), demonstrating that these were homogeneous multicomponent systems. The presence of a singular T_g_ is a key indicator that the components are well-integrated at the molecular level, supporting the conclusion that true coamorphous systems were formed rather than simple physical mixtures of individual amorphous substances.

The experimental and computed T_g_ values provided useful insights into the expected stability of the coamorphous systems. Stability is a critical factor for the practical application of coamorphous materials, particularly because of their inherent tendency to revert to a crystalline state over time or under certain environmental conditions [2,8,36]. It is widely reported in the literature that amorphous and coamorphous systems are generally stable up to a temperature approximately 50 °C below their T_g_, a principle often referred to as the “T_g_—50 °C” rule. According to this rule, molecular mobility within an amorphous solid becomes negligible, 50 °C below its T_g_, whereas temperatures exceeding this threshold can lead to significantly increased molecular mobility, which is sufficient to induce physical changes such as recrystallization or phase separation.

This “T_g_—50 °C” guideline serves as a practical tool for predicting the long-term stability of coamorphous systems, helping to ensure that their amorphous nature is maintained during storage and use [51,52,53]. As a result, coamorphous systems with a T_g_ above 75 °C are generally expected to remain stable under ambient conditions (or even at 40 °C if the T_g_ exceeds 90 °C). In contrast, systems with lower T_g_ values are more prone to recrystallization at room temperature and, therefore, require to be stored in a refrigerator or in a freezer to maintain their physical integrity.

Therefore, to assess the physical stability of the coamorphous systems under different temperature conditions, selected samples (exp. N° 1, 3, 4, 6, 7, 9, and 10) were stored at four distinct temperatures: −30 °C, 5 °C, 25 °C, and 40 °C, over a period of six months. The solid-state nature of these samples was closely monitored using PXRD, with analyses conducted on a weekly basis during the first month and then on a biweekly basis.

**Figure 7:**
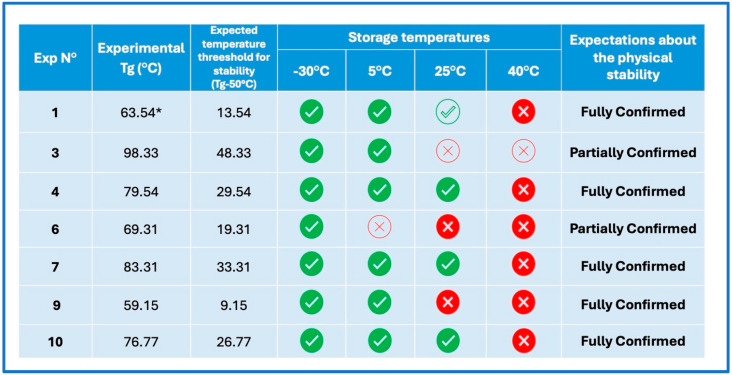
Physical stability of seven coamorphous systems after 6 months of storage at different temperatures. Full sticks indicate that sample meets expectations according to “Tg—50 °C” rule. Full green ticks stand for “stable as expected according to “T_g_—50 °C” rule”; full red crosses stand for “unstable, as expected according to “T_g_—50 °C” rule”; empty green ticks stand for “stability superior to expectations”; empty red crosses stand for “stability inferior to expectations”. * T_g_ value calculated from G-T equation.

Figure 7 presents a summary of the results from this 6-month stability study. The findings largely aligned with the expectations regarding the physical stability of the coamorphous systems, as predicted by the “T_g_—50 °C” rule; only two out of seven systems partially deviated from these expectations. All systems remained stable in the freezer throughout the entire six-month period. Furthermore, one of the samples, specifically exp. N° 1, which corresponds to a ternary coamorphous formulation, for which the T_g_ could not be measured experimentally, considering the “T_g_—50 °C” rule and the calculated T_g_ (through the G-T equation), demonstrated stability greater than expected. This led to conclude with good approximation that this was also a coamorphous system.

**Figure 8:**
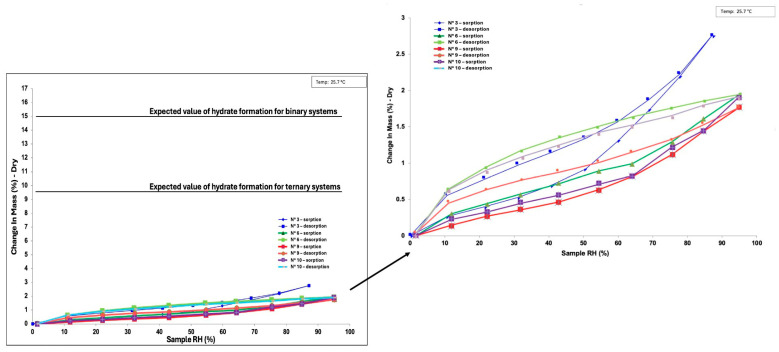
Sorption–desorption curves for exp. N° 3 (dark blue–light blue), 6 (dark green–light green), 9 (red–orange), and 10 (purple–grey).

Remaining in the context of physical stability, one of the most critical challenges associated with amorphous and coamorphous materials is their inherent hygroscopicity. These materials have a high tendency to absorb moisture from the environment due to their disordered molecular structure, which creates an increased surface area and more accessible sites for water interactions. The absorption of moisture can generate several negative consequences. Water molecules, once absorbed, can act as plasticizers, lowering the T_g_ and increasing molecular mobility within the material. This can lead to a destabilization of the amorphous or coamorphous matrix, weakening or completely disrupting the intricate molecular interactions that maintain the material in its high-energy state. Such destabilization brings unwanted phenomena, like phase separation, a loss of cohesion in the coamorphous system, or most detrimentally, recrystallization or hydrates formation. These latter phenomena not only compromise the solubility and dissolution rate advantages of amorphous forms, but also risk reducing the bioavailability [35,36]. Therefore, understanding and meticulously controlling hygroscopicity is crucial to preserving the physical stability of amorphous and coamorphous powders over time. To assess the effects of relative humidity (RH) on the obtained coamorphous systems, selected samples (i.e., N° 3, 6, 9, and 10) were subjected to a gradual RH increase from 0% to 90% at a constant temperature of 25 °C. Figure 8 provides a summary of the sorption–desorption curves for all four coground systems investigated.

In each case, a limited mass uptake can be noticed, not exceeding the 3% of the starting mass. It can be concluded hence that this maximum observed value neither is imputable to a recrystallization event, since after the cycle of desorption the mass corresponds to the starting one, nor to a bulk transformation such as a hydrate formation. In fact, considering the molecular weight of the three APIs and water, a hydrate formation would correspond to a mass gain percentage of about 15% for the binary and 9.6% for the ternary systems. Conversely, a mass uptake ranging around 3% is related to superficial water adsorption, since amorphous phases demonstrate hygroscopic properties and they sorb relatively more water as compared to the crystalline solid phase [54]. A higher level of lattice energy typically results in a less hygroscopic material because the molecules are more tightly bound, making it harder for water to be absorbed. Conversely, lower lattice energy may lead to greater hygroscopicity as the bonds between molecules are weaker and water can more easily be absorbed. The latter is the case of our systems, characterized by a mass uptake of less than 3%, typically related to superficial water adsorption.

The mass uptake of the four samples is between 1.8 and 2.8%, attesting an analogous behavior which does not depend on the composition. Given that all the samples are amorphous, it is not surprising that they exhibit such similar behavior, even though their chemical compositions are sometimes significantly different. Moreover, measurable hysteresis (i.e., a difference in the water vapor uptake between the sorption and desorption isotherms) is visible on the graph, but this are not attributable to any recrystallization phenomena since, as explained above, the final mass after the cycle of desorption corresponds to the initial one.

In addition, it is noteworthy that neither PZQ nor NCM formed hydrated forms during this analysis. This observation is particularly significant given the known tendencies of these compounds to undergo hydration under certain conditions. Specifically, praziquantel readily forms a hemihydrate (indexed as WUHQAW in the CSD [55]) when water is introduced to its amorphous form. Similarly, NCM is highly susceptible to atmospheric humidity, leading to the formation of its monohydrate form (indexed as OBEQAN02 in the CSD [56]), which is notably detrimental due to its extremely low solubility.

Subsequently, drug recovery analyses were carried out on the same seven samples (i.e., exp. N° 1, 3, 4, 6, 7, 9, and 10) used in the physical stability study, focusing on those stored in the freezer at −30 °C and those exposed to stressed conditions at 40 °C (a total of fourteen samples).

Considering that amorphous systems are inherently reactive from a chemical perspective [57] and given that these samples were subjected to an intense milling process lasting 4 h, it was deemed crucial to evaluate their chemical integrity to ensure the quality of a potential pharmaceutical product. This assessment is particularly important as the degradation tendency of PZQ under milling conditions is well-documented in the literature [24,47,58]. While PZQ does not degrade when milled alone [30,31], binary ground mixtures have shown reduced PZQ recovery, along with the emergence of specific degradation products depending on the excipient used [24,47,58]. To determine whether PZQ undergoes chemical degradation when coground with NCM and/or MBZ under NG conditions, spectrometric evaluations were conducted.

The analysis of the recorded ¹H-NMR spectra showed minor signs of PZQ degradation in only one out of fourteen samples (experiment N° 6, a binary coamorphous system). This degradation was likely due to storage at 40 °C, a condition that imposes significant thermal stress and does not reflect standard storage conditions. It is important to note that this study primarily focused on the physical stability of coamorphous systems, specifically to evaluate the validity of the “T_g_—50 °C rule,” rather than serving as a comprehensive stability assessment for drugs or medicinal products in alignment with EMA guidelines or international standards such as the ICH Q1A (R2) guidelines [59].

Encouragingly, all other thirteen samples, including those stored at 40 °C, showed only the presence of the starting materials—PZQ, NCM, and/or MBZ—indicating the absence of chemical changes after mechanochemical treatment under all experimental conditions. Figures S23–S36 in the SI file report all the recorded ¹H-NMR spectra.

The final phase of this experimental work involved clarifying the recrystallization phenomena observed in the newly developed systems.

The recrystallization of coamorphous systems can lead to different outcomes, depending on the nature of the components and their interactions. In some cases, recrystallization may result in the separation of the initial phases, where each component reverts to its crystalline form independently. Alternatively, recrystallization can lead to the formation of a new entity that corresponds to the coamorphous system but in a crystalline form, known as a cocrystal. This transformation into a cocrystal indicates that the components have not only maintained their interactions, but have rearranged into a well-defined crystalline lattice, preserving the combined properties of the coamorphous phase while gaining the stability of a crystalline structure [60,61,62].

Here, we present two examples of recrystallization pathways, noticed during stability studies: one illustrating the recrystallization of a coamorphous system into its individual components, and the other showcasing its transformation into a new cocrystal. In some cases, to gain a deeper understanding of the solid forms obtained after recrystallization, SEM images of both the fresh and recrystallized products were acquired and combined with PXRD analyses (see Figure S37 in the SI file as an example).

**Figure 9:**
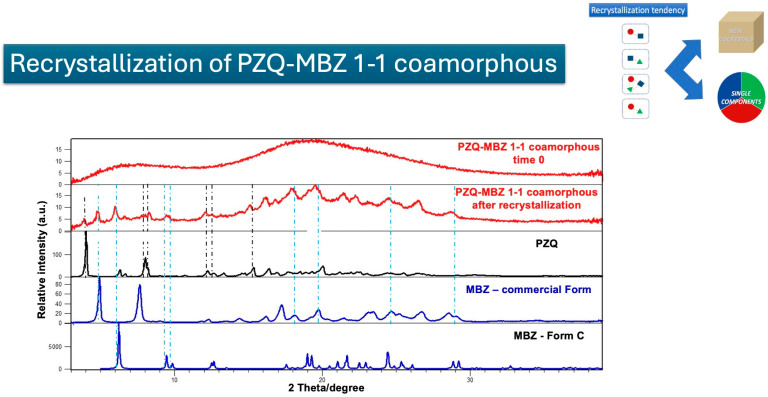
Cartoon depicting the recrystallization of binary PZQ-MBZ 1–1 coamorphous systems into individual components through PXRD analyses. Black dotted lines represent PZQ peaks, while light blue dotted lines MBZ reflections.

The first example of recrystallization is that observed in exp. N° 6, which corresponds to the binary PZQ-MBZ 1–1 coamorphous system. As shown in Figure 9, which presents the PXRD data of the fresh and recrystallized products, the recrystallization process leads to the phase separation of the original components, PZQ and MBZ. In fact, the PXRD patterns clearly show distinct peaks corresponding to the separate crystalline forms of PZQ and MBZ (as a mixture of Polymorphs A and C).

The second example is exp. N° 9, which involves the binary PZQ-NCM 1–1 coamorphous system. In this instance, recrystallization did not result in the formation of individual crystalline materials. Instead, it led to the creation of a “new” entity that, through a detailed analysis, was identified as the previously reported PZQ-NCM 1–3 anhydrous cocrystal (CSD refcode RIPFOP01) [26].

**Figure 10:**
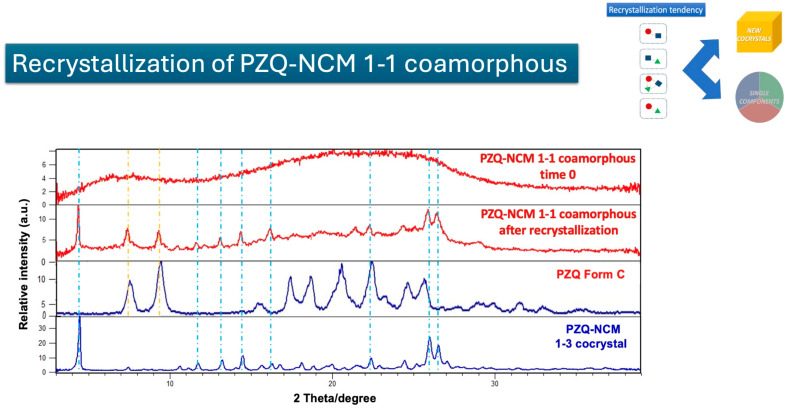
Cartoon depicting the recrystallization of binary PZQ-NCM 1–1 coamorphous system into polymorph C of PZQ and the PZQ-NCM 1–3 anhydrous cocrystal. Yellow dotted lines represent PZQ polymorph C reflections, while light blue dotted lines PZQ-NCM 1–3 anhydrous cocrystal reflections.

What is particularly intriguing about this coamorphous system is its unique behavior during recrystallization. Specifically, the PZQ-NCM 1–1 coamorphous system transforms into a combination of the well-known polymorph C of PZQ and PZQ-NCM 1–3 anhydrous cocrystal (Figure 10). This indicates a significant change in the stoichiometry between the two components, suggesting that the coamorphous state can influence the pathways of crystallization in unexpected ways.

However, recrystallization phenomena are inherently slow processes, and understanding them, particularly in ternary coground systems, can often be challenging and not immediately straightforward. Due to the complexity of these systems and the gradual nature of recrystallization, this aspect of the research is still in progress and continues to expand.

The results of this scientific work, particularly those related to the physical stability of co-amorphous systems, highlight a significant advancement achieved through the development of ternary co-amorphous systems. These systems have demonstrated superior physical stability over binary systems, representing a key innovation of this study. This enhanced stability can be attributed to the peculiarity of ternary systems compared to binary counterparts. In binary systems, one drug often serves as a coformer for the other, limiting the interaction network to primarily two components. In contrast, ternary coamorphous systems leverage the inclusion of a carefully selected third component, which introduces additional molecular complexity. This third component contributes to a more intricate and robust interaction network that strengthens the stability of the amorphous state. The scientific literature supports the notion that the addition of a third component facilitates stronger and more diverse molecular interactions, such as hydrogen bonding, dipole–dipole interactions, and van der Waals forces. These interactions collectively inhibit recrystallization processes and improve the physical stability of the system [7,12,13]. In this study, experiments involving ternary coamorphous systems have demonstrated that the introduction of a third component not only broadens the interaction spectrum but can also increase the system glass transition temperature (T_g_), thus reducing molecular mobility and guaranteeing prolonged stability [57,63].

In conclusion, the innovation of ternary coamorphous systems lies in their ability to combine molecular complexity with stronger interaction networks, thereby surpassing the physical stability of binary systems. Future research should further explore a broader range of pharmaceutical compounds to fully harness the potential of ternary systems.

## 4. Conclusions

In this experimental work, we demonstrate the possibility of preparing coamorphous systems composed exclusively of active pharmaceutical ingredients, specifically praziquantel, niclosamide, and mebendazole.

The coamorphous systems were prepared via a sustainable one-step process—neat grinding—using a lab-scale vibrational mill. The process proved to be reproducible, requiring only 4 h of grinding to achieve the coamorphous state. All systems were characterized using PXRD, with some samples additionally analyzed through FT-IR. These results were compared with the starting components and their corresponding physical mixtures to confirm the absence of crystalline domains in the samples and to investigate the interactions between the components. Glass transition temperatures of the coamorphous systems were experimentally determined using DSC and compared to theoretical T_g_ values calculated via the Gordon–Taylor equation. Despite the complex DSC curves, particularly for ternary coamorphous systems, the presence of a single T_g_ in each case confirmed the formation of a homogeneous multicomponent amorphous phase.

Stability studies were conducted on seven systems (three binary and four ternary coground samples) for six months under four different temperature conditions (−30 °C, 5 °C, 25 °C, and 40 °C), based on the “T_g_—50 °C” stability rule. The results largely aligned with expectations for physical stability, with only two deviations out of the seven systems. All systems remained stable when stored in the freezer for the entire six-month period, and one ternary coamorphous formulation exhibited a higher stability temperature than anticipated.

Regarding the influence of the relative humidity, preliminary DVS results indicated that water had a minimal impact on the new solid phases in a short period. Only superficial sorption was observed, with no evidence of humidity-induced recrystallization or solid hydrate formation.

Recrystallization pathways were also investigated to better understand the potential outcomes of this process. Depending on the molecular interactions within the system, recrystallization led either to the separation of the initial components into their crystalline forms or to the formation of a new entity, such as a cocrystal previously reported by our group.

Future directions of this work will focus on a deeper examination of the recrystallization phenomena, which are slow processes, particularly in ternary systems. Additionally, the biopharmaceutical properties of these promising drug–drug coamorphous systems—such as solubility, dissolution, permeability, and their anthelmintic activity—will be investigated to further evaluate their therapeutic potential.

## Figures and Tables

**Table 1:** Experimental matrix.

Exp. N°	Molar Ratio		
	PZQ X1	MBZ X2	NCM X3
**1**	0.500	0.250	0.250
**2**	0.167	0.417	0.417
**3**	0	0.500	0.500
**4**	0.250	0.500	0.250
**5**	0.417	0.167	0.417
**6**	0.500	0	0.500
**7**	0.250	0.250	0.500
**8**	0.417	0.417	0.167
**9**	0.500	0.500	0
**10**	0.333	0.333	0.333

**Table 2:** Experimental T_g_ values compared to theoretical ones according to G-T equation.

Sample/Exp. N°	Molar Ratio			Experimental T_g_ (°C) ± S.D.	Theoretical T_g_ According to G-T Equation (°C)
	PZQ	NCM	MBZ		
**PZQ**	1	0	0	40.94 * ± 0.90	28.56 ^§^
**NCM**	0	1	0	82.90 * ± 0.64	85.47 ^§^
**MBZ**	0	0	1	111.98 ± 0.73	142.95 ^§^
**1**	0.500	0.250	0.250	/	63.54
**2**	0.167	0.417	0.417	/	84.17
**3**	0	0.500	0.500	98.33 ± 0.34	96.95
**4**	0.250	0.500	0.250	79.54 ± 0.59	75.40
**5**	0.417	0.167	0.417	74.02 ± 0.59	71.02
**6**	0.500	0	0.500	69.31 ± 0.87	68.67
**7**	0.250	0.250	0.500	83.31 ± 0.76	81.58
**8**	0.417	0.417	0.167	60.40 ± 0.13	65.40
**9**	0.500	0.500	0	59.15 ± 0.95	57.87
**10**	0.333	0.333	0.333	76.77 ± 0.09	73.15

* MTDSC. ^§^ Single compound, not applicable to calculate a theoretical T_g_ according to G-T equation; T_g_ was predicted through the model proposed by Alzghoul et al. [45].

## Data Availability

The original contributions presented in this study are included in the article/Supplementary Materials. Further inquiries can be directed to the corresponding author.

## Supplementary Materials

The following supporting information can be downloaded at: https://www.mdpi.com/article/10.3390/pharmaceutics17010092/s1, Figure S1. PXRD analysis of 4 repetitions of NG of exp. N° 1 (ternary PZQ-NCM-MBZ 1–0.5–0.5 system). Figure S2. PXRD analysis of 4 repetitions of NG of exp. N° 2 (ternary PZQ-NCM-MBZ 1–2.5–2.5 system). Figure S3. PXRD analysis of 8 repetitions of NG of exp. N° 3 (binary NCM-MBZ 1–1 system). Figure S4. PXRD analysis of 8 repetitions of NG of exp. N° 4 (ternary PZQ-NCM-MBZ 0.5–0.5–1 system). Figure S5. PXRD analysis of 4 repetitions of NG of exp. N° 5 (ternary PZQ-NCM-MBZ 2.5–2.5–1 system). Figure S6. PXRD analysis of 6 repetitions of NG of exp. N° 6 (binary PZQ-NCM 1–1 system). Figure S7. PXRD analysis of 4 repetitions of NG of exp. N° 7 (ternary PZQ-NCM-MBZ 0.5−1−0.5 system). Figure S8. PXRD analysis of 4 repetitions of NG of exp. N° 8 (ternary PZQ-NCM-MBZ 2.5−1−2.5 system). Figure S9. PXRD analysis of 8 repetitions of NG of exp. N° 9 (binary PZQ-MBZ 1−1 system). Figure S10. PXRD analysis of 6 repetitions of NG of exp. N° 10 (ternary PZQ-NCM-MBZ 1−1−1 system). Figure S11. PXRD analysis of pure MBZ, PZQ, and NCM compared to the physical mixtures of the matrix systems. Figure S12. DSC curve showing T_g_ of pure PZQ. Figure S13. DSC curve showing T_g_ of pure NCM. Figure S14. DSC curve showing T_g_ of pure MBZ. Figure S15. DSC curve showing T_g_ of exp. N° 3 (binary NCM-MBZ 1-1 system). Figure S16. DSC curve showing T_g_ of exp. N° 4 (ternary PZQ-NCM-MBZ 0.5-0.5-1 system). Figure S17. DSC curve showing T_g_ of exp. N° 5 (ternary PZQ-NCM-MBZ 2.5-1-2.5 system). Figure S18. DSC curve showing T_g_ of exp. N° 6 (binary PZQ-NCM 1-1 system). Figure S19. DSC curve showing T_g_ of exp. N° 7 (ternary PZQ-NCM-MBZ 0.5-1-0.5 system). Figure S20. DSC curve showing T_g_ of exp. N° 8 (ternary PZQ-NCM-MBZ 2.5-2.5-1 system). Figure S21. DSC curve showing T_g_ of exp. N° 9 (binary PZQ-MBZ 1-1 system). Figure S22. DSC curve showing T_g_ of exp. N° 10 (ternary PZQ-NCM-MBZ 1-1-1 system). Figure S23. ^1^H-NMR analysis (from top to bottom) of pure PZQ, NCM, and MBZ compared to the sample of exp. N° 1. Figure S24. ^1^H-NMR analysis (from top to bottom) of pure PZQ, NCM, and MBZ compared to the sample of exp. N° 2. Figure S25. ^1^H-NMR analysis (from top to bottom) of pure PZQ, NCM, and MBZ compared to the sample of exp. N° 3. Figure S26. ^1^H-NMR analysis (from top to bottom) of pure PZQ, NCM, and MBZ compared to the sample of exp. N° 4. Figure S27. ^1^H-NMR analysis (from top to bottom) of pure PZQ, NCM, and MBZ compared to the sample of exp. N° 5. Figure S28. ^1^H-NMR analysis (from top to bottom) of pure PZQ, NCM, and MBZ compared to the sample of exp. N° 6. Figure S29. ^1^H-NMR analysis (from top to bottom) of pure PZQ, NCM, and MBZ compared to the sample of exp. N° 7. Figure S30. ^1^H-NMR analysis (from top to bottom) of pure PZQ, NCM, and MBZ compared to the sample of exp. N° 8. Figure S31. ^1^H-NMR analysis (from top to bottom) of pure PZQ, NCM, and MBZ compared to the sample of exp. N° 9. Figure S32. ^1^H-NMR analysis (from top to bottom) of pure PZQ, NCM, and MBZ compared to the sample of exp. N° 10. Figure S33. ^1^H-NMR analysis (from top to bottom) of pure PZQ, NCM, and MBZ compared to the sample of exp. N° 11. Figure S34. ^1^H-NMR analysis (from top to bottom) of pure PZQ, NCM, and MBZ compared to the sample of exp. N° 12. Figure S35. ^1^H-NMR analysis (from top to bottom) of pure PZQ, NCM, and MBZ compared to the sample of exp. N° 13. Figure S36. ^1^H-NMR analysis (from top to bottom) of pure PZQ, NCM, and MBZ compared to the sample of exp. N° 14. Figure S37. Cartoon depicting the recrystallization of ternary PZQ-MBZ-NCM 1-1-1 coamorphous into MBZ and the PZQ-NCM 1-3 anhydrous cocrystal through SEM and PXRD. Black dotted lines represent MBZ reflections, while light blue dotted lines PZQ-NCM 1-3 anhydrous cocrystal reflections.
