# Supplementary material for: Stability of Ternary Drug–Drug–Drug Coamorphous Systems Obtained Through Mechanochemistry
Source: Pharmaceutics. 2025 Jan 12;17(1):92. doi: 10.3390/pharmaceutics17010092 (PMC11769221; doi:10.3390/pharmaceutics17010092)
Supplement: Supplementary file 1 [file pharmaceutics-17-00092-s001.zip › pharmaceutics-3385733-supplementary.pdf]

Supporting information

Stability of Ternary Drug–Drug–Drug Coamorphous Systems Obtained Through Mechanochemistry

Ilenia D'Abbrunzo [1], Elisabetta Venier [1], Francesca Selmin [2], Irena Škorić [3], Enrico Bernardo [4], Giuseppe Procida [1] and Beatrice Perissutti [1,*]

[1] Department of Chemical and Pharmaceutical Sciences, University of Trieste, Piazzale Europa 1, 34127 Trieste, Italy; ilenia.d'abbrunzo@phd.units.it (I.D.); elisabetta.venier@studenti.units.it (E.V.); gprocida@units.it (G.P.)
[2] Department of Pharmaceutical Sciences, University of Milan, Via G. Colombo, 71, 20133 Milan, Italy; francesca.selmin@unimi.it
[3] Department of Organic Chemistry, Faculty of Chemical Engineering and Technology, University of Zagreb, Marulićev trg 19, 10000 Zagreb, Croatia; iskoric@fkit.unizg.hr
[4] Department of Industrial Engineering, University of Padova, Via F. Marzolo 9, 35131 Padova, Italy; enrico.bernardo@unipd.it
* Correspondence: bperissutti@units.it

Contents

**Description**	**Page N°**
**Figure S1.** PXRD analysis of 4 repetitions of NG of exp. N° 1 (ternary PZQ-NCM-MBZ 1-0.5-0.5 system).	**3**
**Figure S2.** PXRD analysis of 4 repetitions of NG of exp. N° 2 (ternary PZQ-NCM-MBZ 1-2.5-2.5 system).	**3**
**Figure S3.** PXRD analysis of 8 repetitions of NG of exp. N° 3 (binary NCM-MBZ 1-1 system).	**4**
**Figure S4.** PXRD analysis of 8 repetitions of NG of exp. N° 4 (ternary PZQ-NCM-MBZ 0.5-0.5-1 system).	**4**
**Figure S5.** PXRD analysis of 4 repetitions of NG of exp. N° 5 (ternary PZQ-NCM-MBZ 2.5-2.5-1 system).	**5**
**Figure S6.** PXRD analysis of 6 repetitions of NG of exp. N° 6 (binary PZQ-NCM 1-1 system).	**5**
**Figure S7.** PXRD analysis of 4 repetitions of NG of exp. N° 7 (ternary PZQ-NCM-MBZ 0.5-1-0.5 system).	**6**
**Figure S8.** PXRD analysis of 4 repetitions of NG of exp. N° 8 (ternary PZQ-NCM-MBZ 2.5-1-2.5 system).	**6**
**Figure S9.** PXRD analysis of 8 repetitions of NG of exp. N° 9 (binary PZQ-MBZ 1-1 system).	**7**
**Figure S10.** PXRD analysis of 6 repetitions of NG of exp. N° 10 (ternary PZQ-NCM-MBZ 1-1-1 system).	**7**
**Figure S11.** PXRD analysis of pure MBZ, PZQ and NCM compared to the physical mixtures of the matrix systems.	**8**
**Figure S12.** DSC curve showing T_g of pure PZQ.	**8**
**Figure S13.** DSC curve showing T_g of pure NCM.	**9**
**Figure S14.** DSC curve showing T_g of pure MBZ.	**9**
**Figure S15.** DSC curve showing T_g of exp. N° 3 (binary NCM-MBZ 1-1 system).	**10**
**Figure S16.** DSC curve showing T_g of exp. N° 4 (ternary PZQ-NCM-MBZ 0.5-0.5-1 system).	**10**
**Figure S17.** DSC curve showing T_g of exp. N° 5 (ternary PZQ-NCM-MBZ 2.5-1-2.5 system).	**11**
**Figure S18.** DSC curve showing T_g of exp. N° 6 (binary PZQ-NCM 1-1 system).	**11**
**Figure S19.** DSC curve showing T_g of exp. N° 7 (ternary PZQ-NCM-MBZ 0.5-1-0.5 system).	**12**

**Figure S20.** DSC curve showing T_g of exp. N° 8 (ternary PZQ-NCM-MBZ 2.5-2.5-1 system).	**12**
**Figure S21.** DSC curve showing T_g of exp. N° 9 (binary PZQ-MBZ 1-1 system).	**13**
**Figure S22.** DSC curve showing T_g of exp. N° 10 (ternary PZQ-NCM-MBZ 1-1-1 system).	**13**
**Figure S23.** ^{1}H-NMR analysis (from top to bottom) of pure PZQ, NCM and MBZ compared to the sample of exp. N° 1	**14**
**Figure S24.** ^{1}H-NMR analysis (from top to bottom) of pure PZQ, NCM and MBZ compared to the sample of exp. N° 2.	**14**
**Figure S25.** ^{1}H-NMR analysis (from top to bottom) of pure PZQ, NCM and MBZ compared to the sample of exp. N° 3.	**14**
**Figure S26.** ^{1}H-NMR analysis (from top to bottom) of pure PZQ, NCM and MBZ compared to the sample of exp. N° 4.	**15**
**Figure S27.** ^{1}H-NMR analysis (from top to bottom) of pure PZQ, NCM and MBZ compared to the sample of exp. N° 5.	**15**
**Figure S28.** ^{1}H-NMR analysis (from top to bottom) of pure PZQ, NCM and MBZ compared to the sample of exp. N° 6	**15**
**Figure S29.** ^{1}H-NMR analysis (from top to bottom) of pure PZQ, NCM and MBZ compared to the sample of exp. N° 7.	**16**
**Figure S30.** ^{1}H-NMR analysis (from top to bottom) of pure PZQ, NCM and MBZ compared to the sample of exp. N° 8	**16**
**Figure S31.** ^{1}H-NMR analysis (from top to bottom) of pure PZQ, NCM and MBZ compared to the sample of exp. N° 9	**16**
**Figure S32.** ^{1}H-NMR analysis (from top to bottom) of pure PZQ, NCM and MBZ compared to the sample of exp. N° 10	**17**
**Figure S33.** ^{1}H-NMR analysis (from top to bottom) of pure PZQ, NCM and MBZ compared to the sample of exp. N° 11	**17**
**Figure S34.** ^{1}H-NMR analysis (from top to bottom) of pure PZQ, NCM and MBZ compared to the sample of exp. N° 12.	**17**
**Figure S35.** ^{1}H-NMR analysis (from top to bottom) of pure PZQ, NCM and MBZ compared to the sample of exp. N° 13.	**18**
**Figure S36.** ^{1}H-NMR analysis (from top to bottom) of pure PZQ, NCM and MBZ compared to the sample of exp. N° 14.	**18**
**Figure S37.** Cartoon depicting the recrystallization of ternary PZQ-MBZ-NCM 1-1-1 coamorphous into MBZ and the PZQ-NCM 1-3 anhydrous cocrystal through SEM and PXRD. Black dotted lines represent MBZ reflections, while light blue dotted lines PZQ-NCM 1-3 anhydrous cocrystal reflections.	**19**

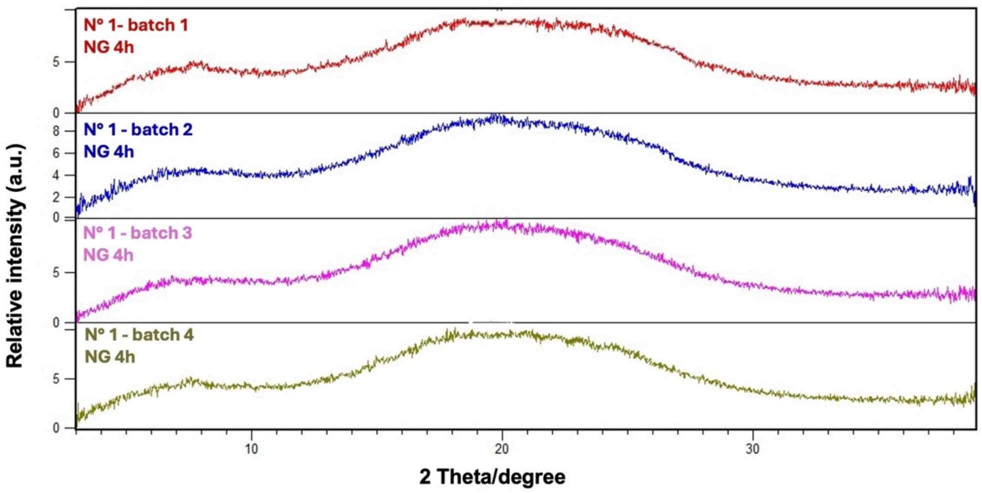

Figure S1. PXRD analysis of 4 repetitions of NG of exp. N° 1 (ternary PZQ-NCM-MBZ 1-0.5-0.5 system).

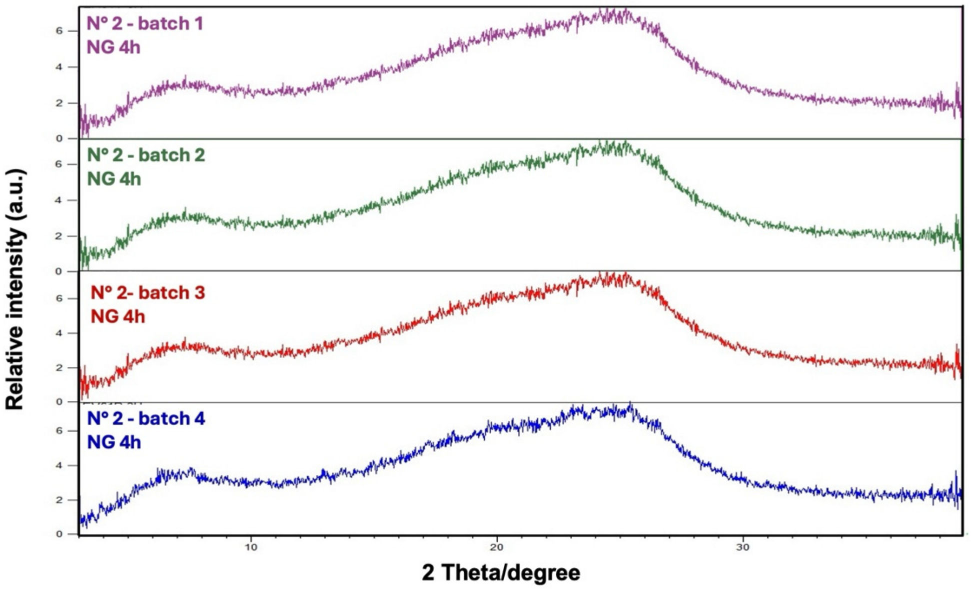

Figure S2. PXRD analysis of 4 repetitions of NG of exp. N° 2 (ternary PZQ-NCM-MBZ 1-2.5-2.5 system).

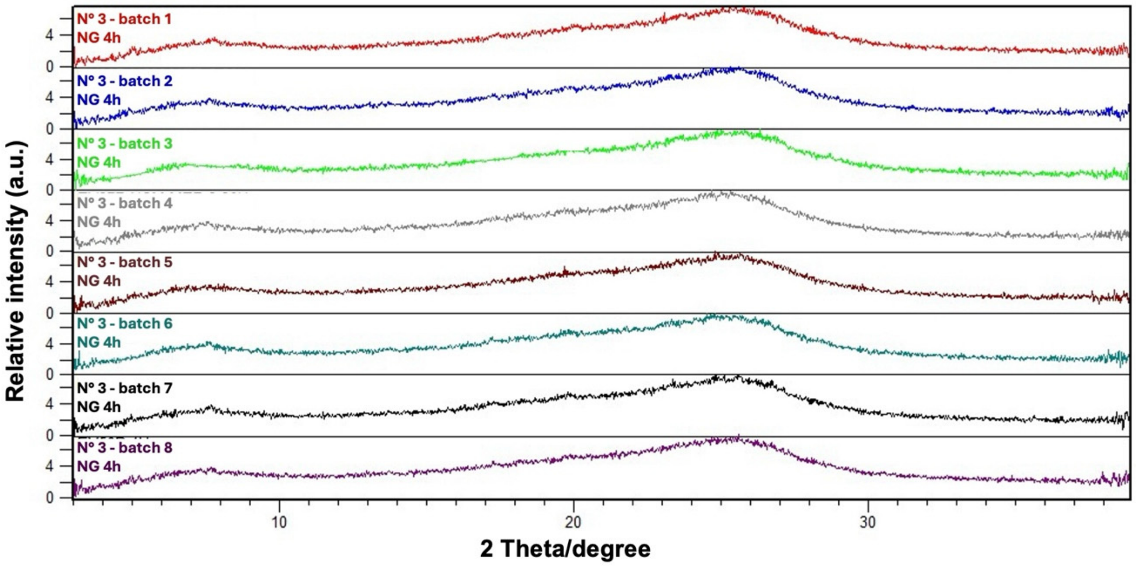

Figure S3. PXRD analysis of 8 repetitions of NG of exp. N° 3 (binary NCM-MBZ 1-1 system).

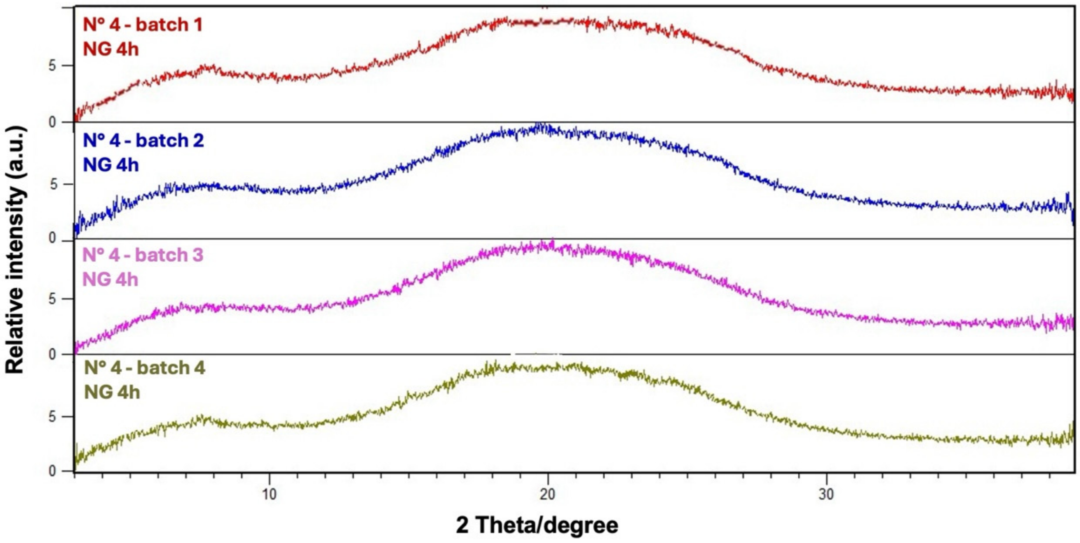

Figure S4. PXRD analysis of 8 repetitions of NG of exp. N° 4 (ternary PZQ-NCM-MBZ 0.5-0.5-1 system).

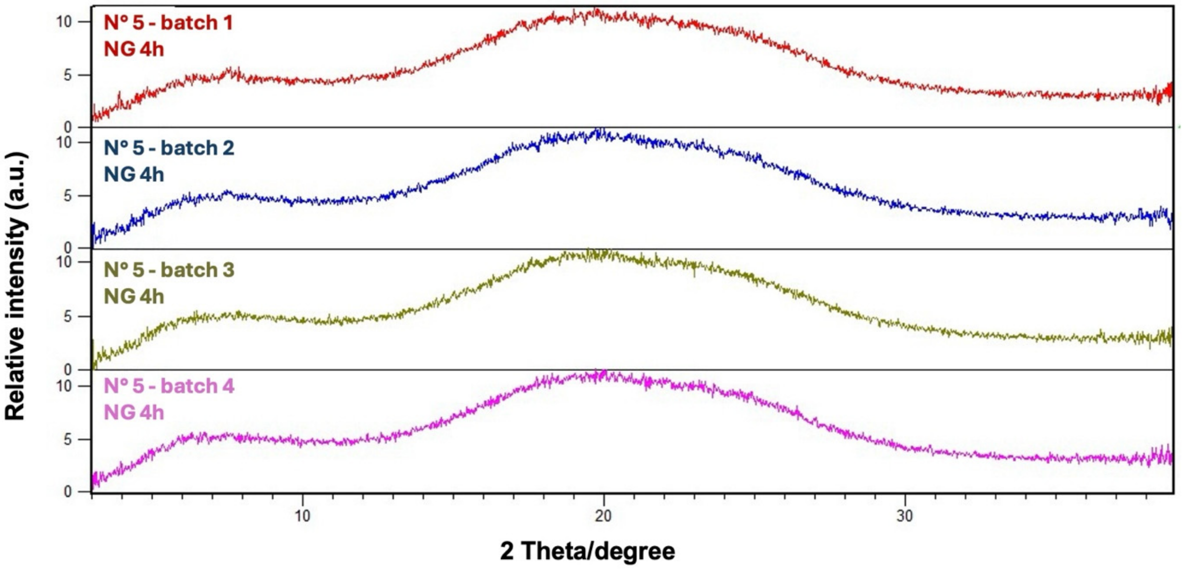

Figure S5. PXRD analysis of 4 repetitions of NG of exp. N° 5 (ternary PZQ-NCM-MBZ 2.5-1-2.5 system).

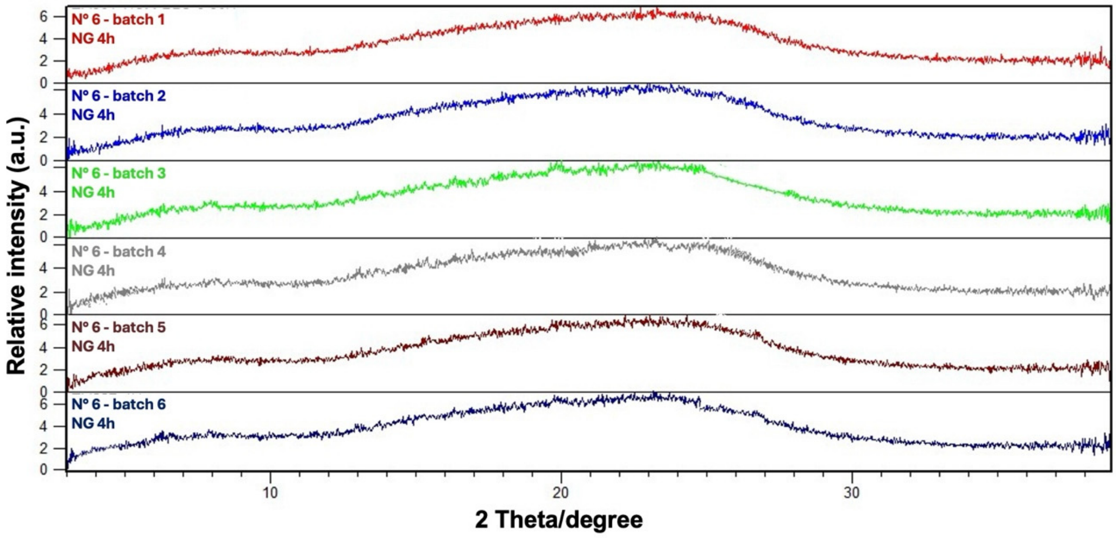

Figure S6. PXRD analysis of 6 repetitions of NG of exp. N° 6 (binary PZQ-NCM 1-1 system).

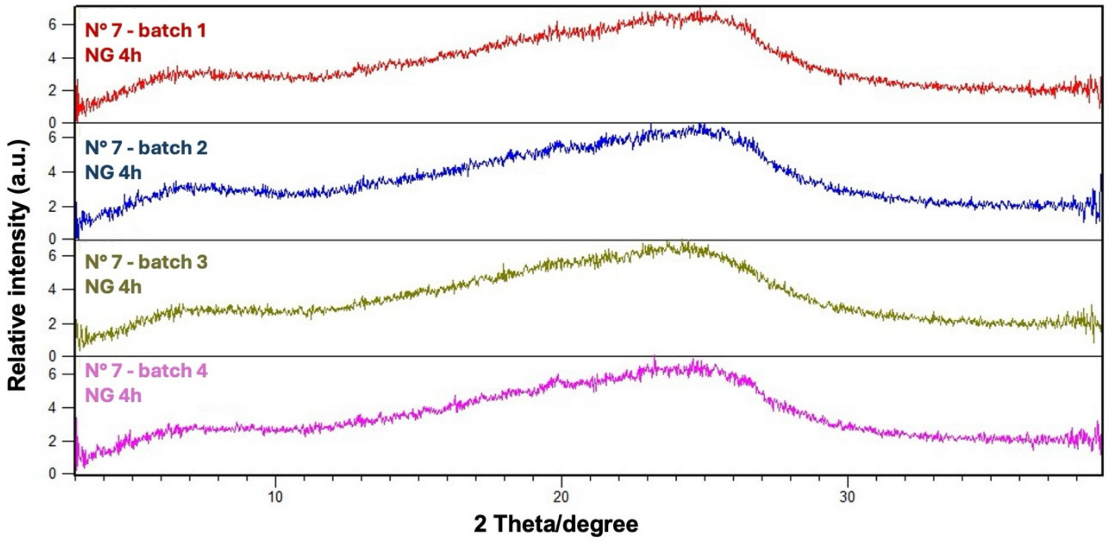

Figure S7. PXRD analysis of 4 repetitions of NG of exp. N° 7 (ternary PZQ-NCM-MBZ 0.5-1-0.5 system).

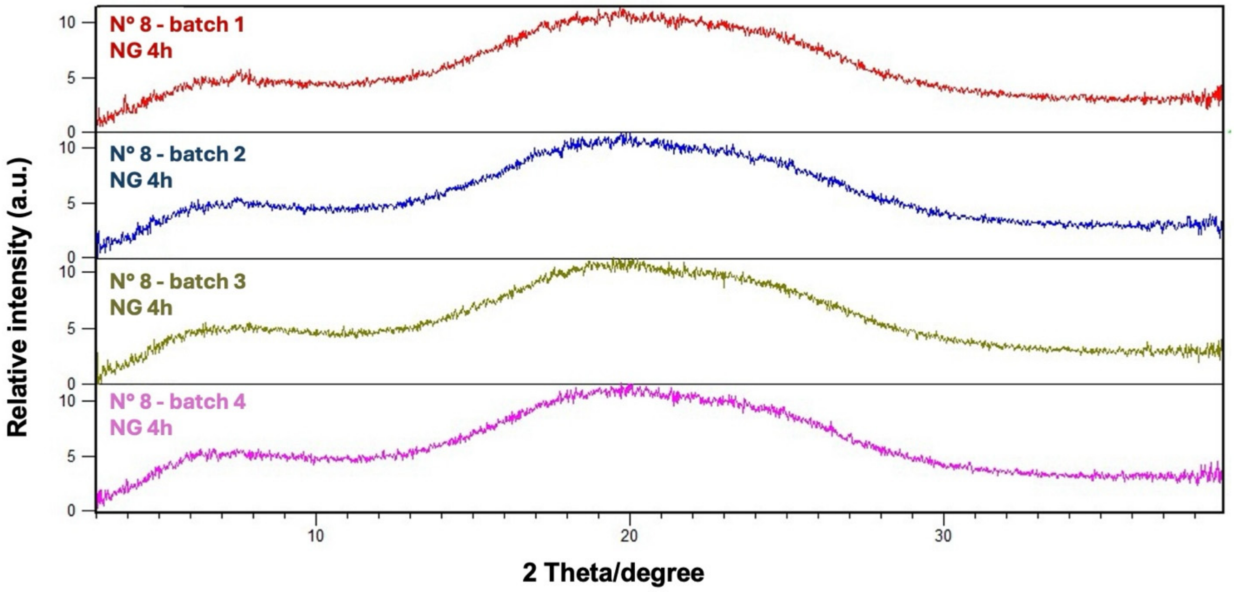

Figure S8. PXRD analysis of 4 repetitions of NG of exp. N° 8 (ternary PZQ-NCM-MBZ 2.5-2.5-1 system).

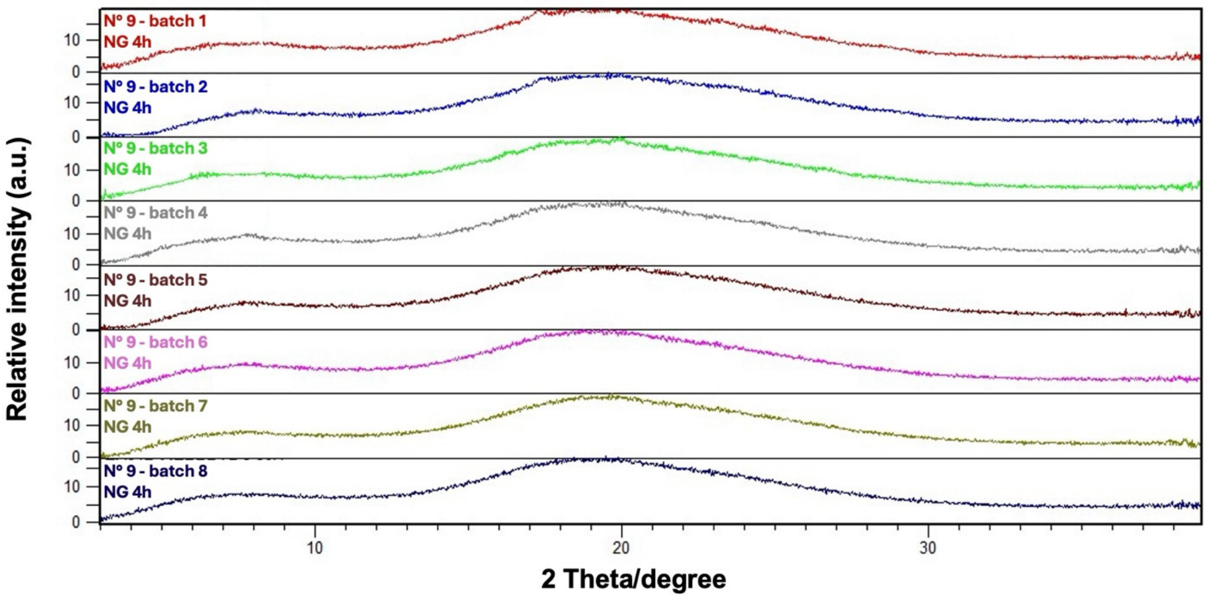

Figure S9. PXRD analysis of 8 repetitions of NG of exp. N° 9 (binary PZQ-MBZ 1-1 system).

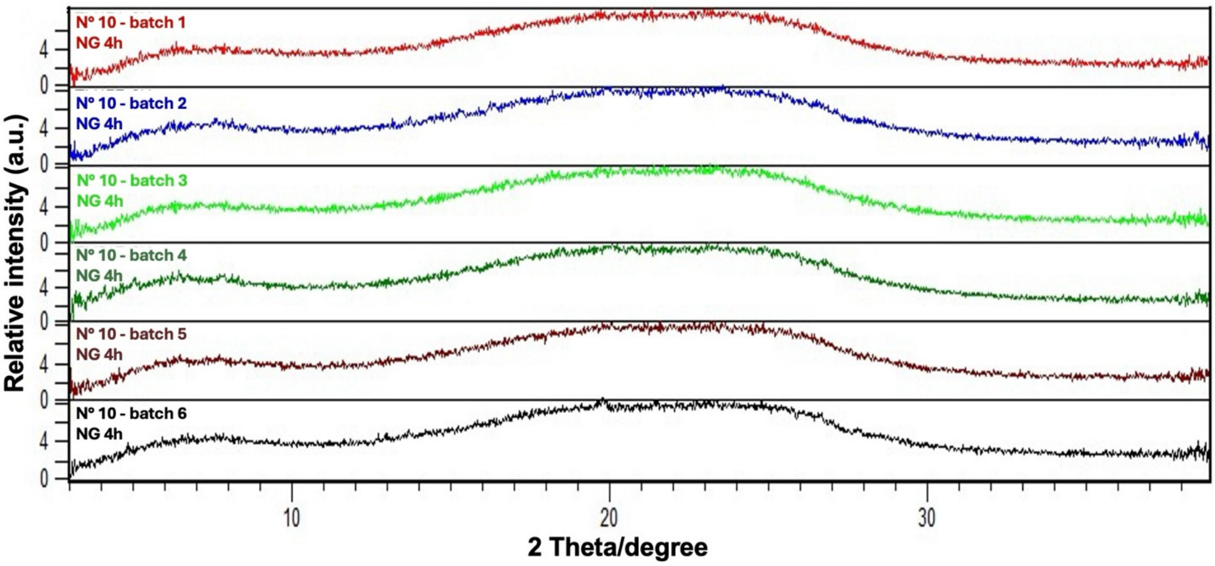

Figure S10. PXRD analysis of 6 repetitions of NG of exp. N° 10 (ternary PZQ-NCM-MBZ 1-1-1 system).

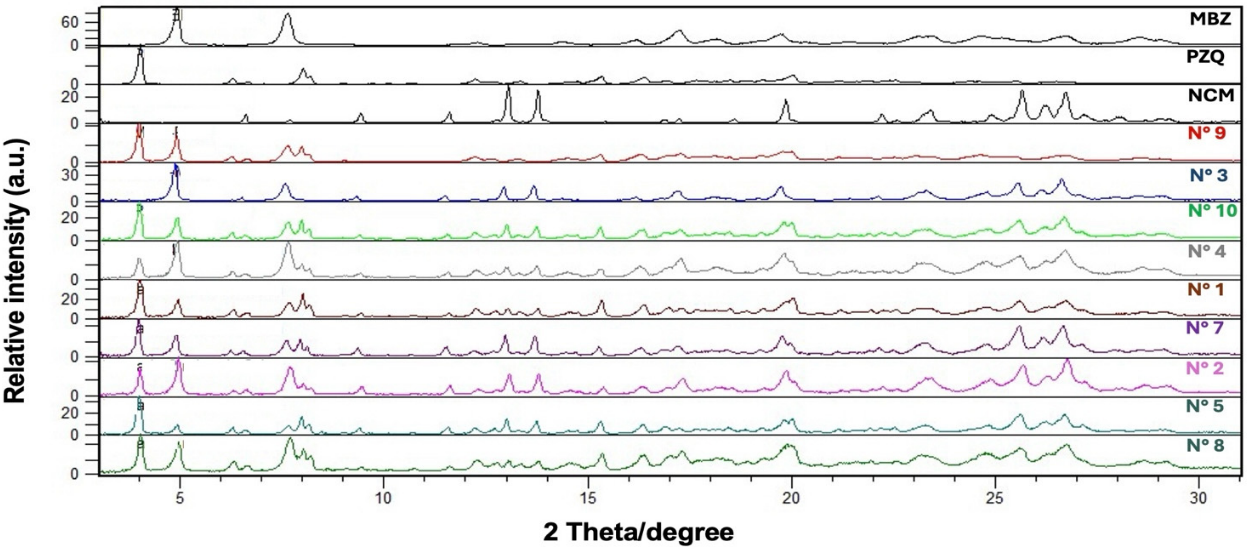

Figure S11. PXRD analysis of pure MBZ, PZQ and NCM compared to the physical mixtures of the matrix systems.

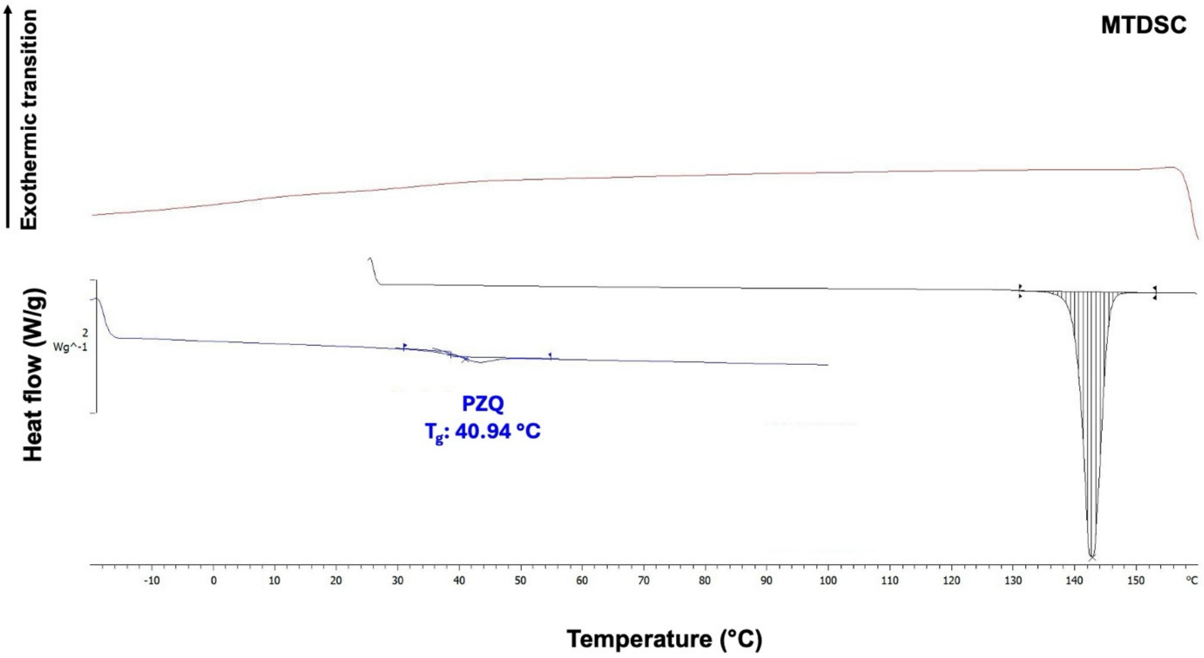

Figure S12. DSC curve showing T_g of pure PZQ.

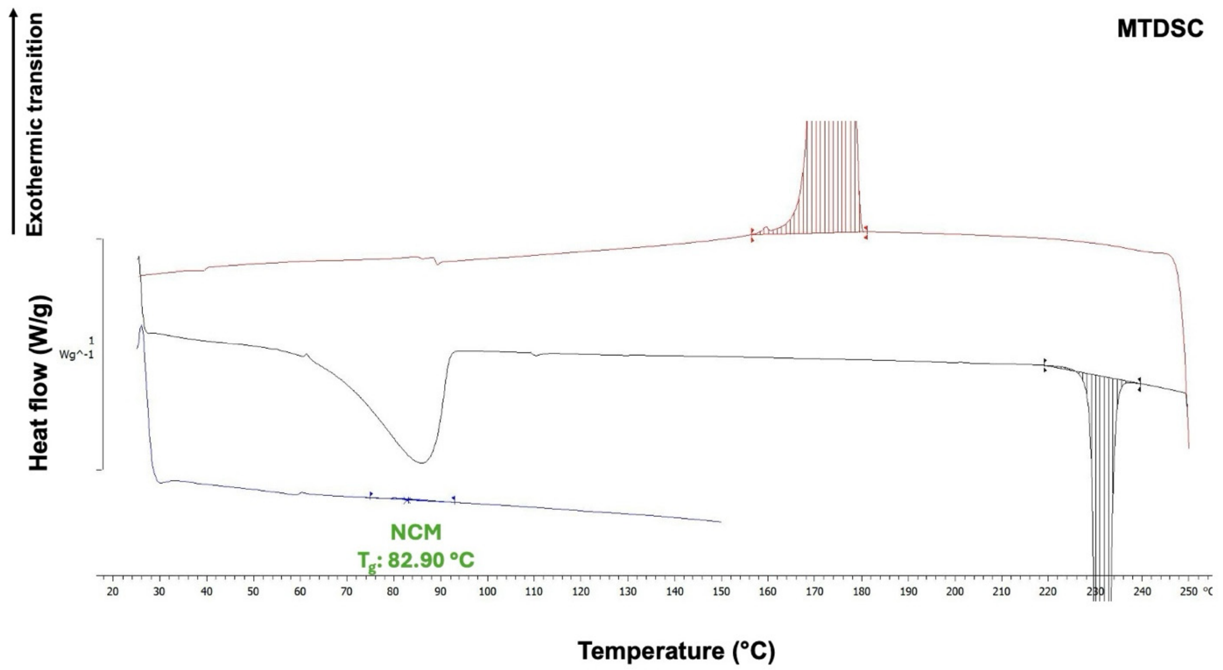

Figure S13. DSC curve showing T_g of pure NCM.

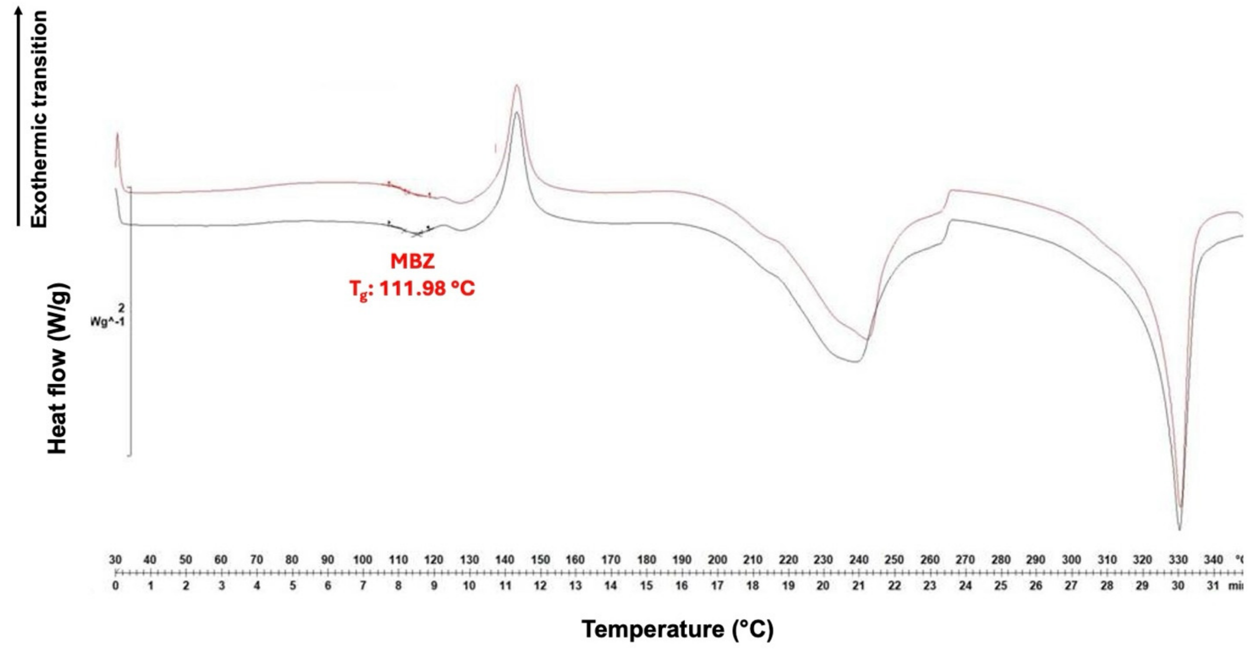

Figure S14. DSC curve showing T_g of pure MBZ.

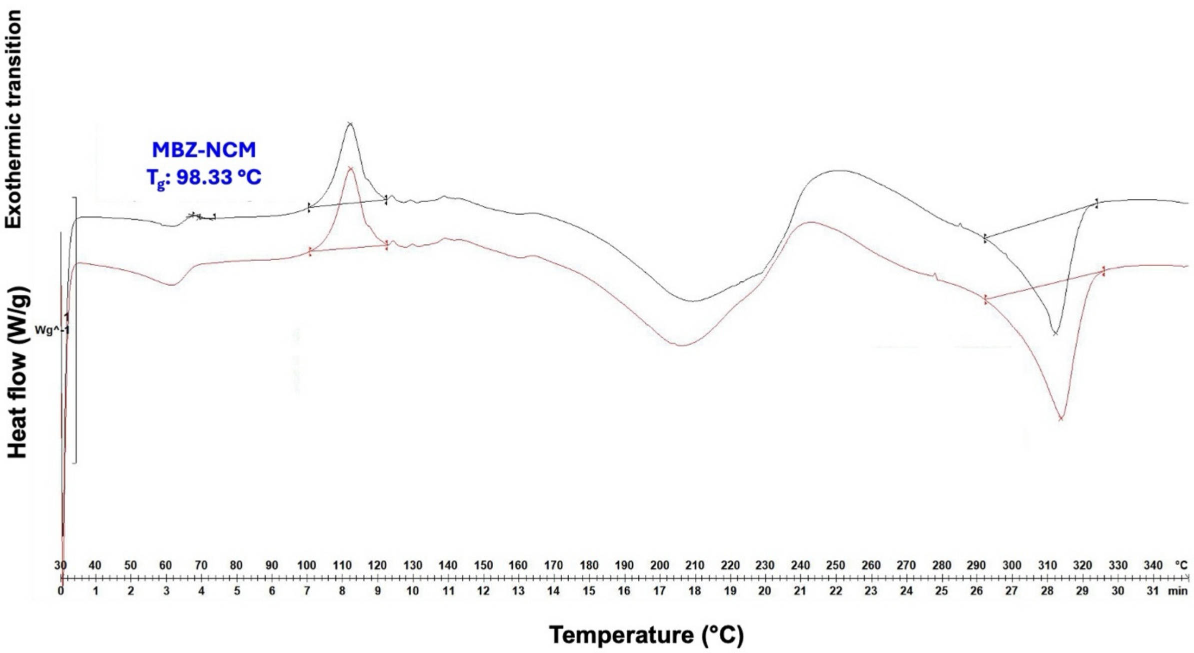

Figure S15. DSC curve showing T_g of exp. N° 3 (binary NCM-MBZ 1-1 system).

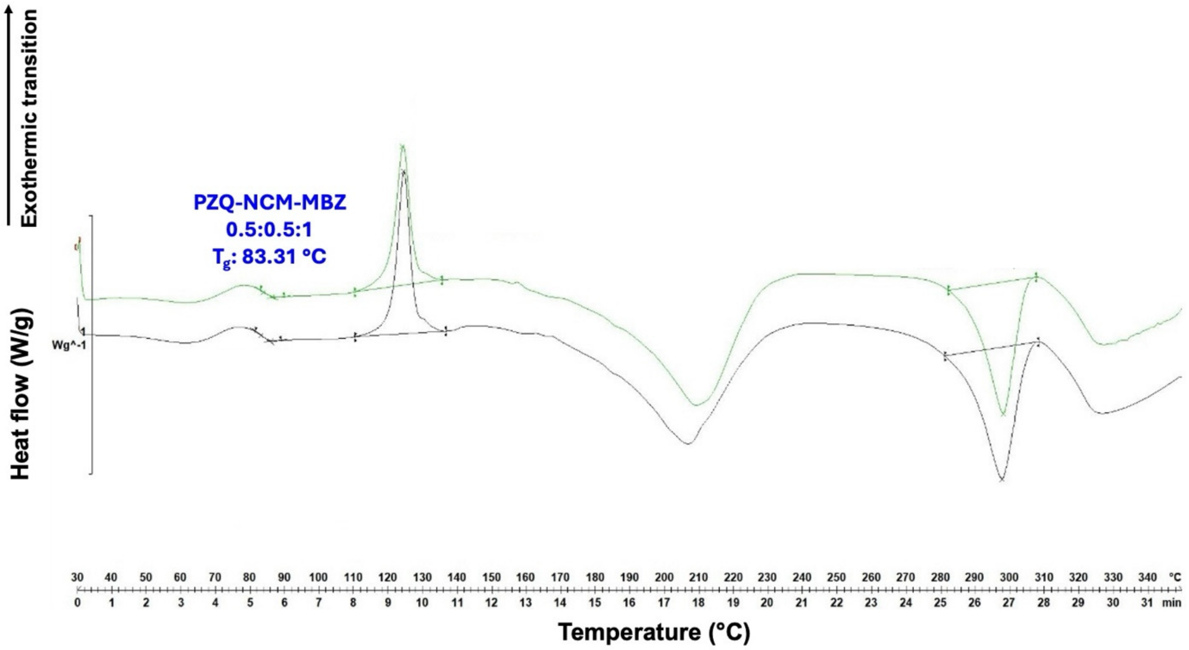

Figure S16. DSC curve showing T_g of exp. N° 4 (ternary PZQ-NCM-MBZ 0.5-0.5-1 system).

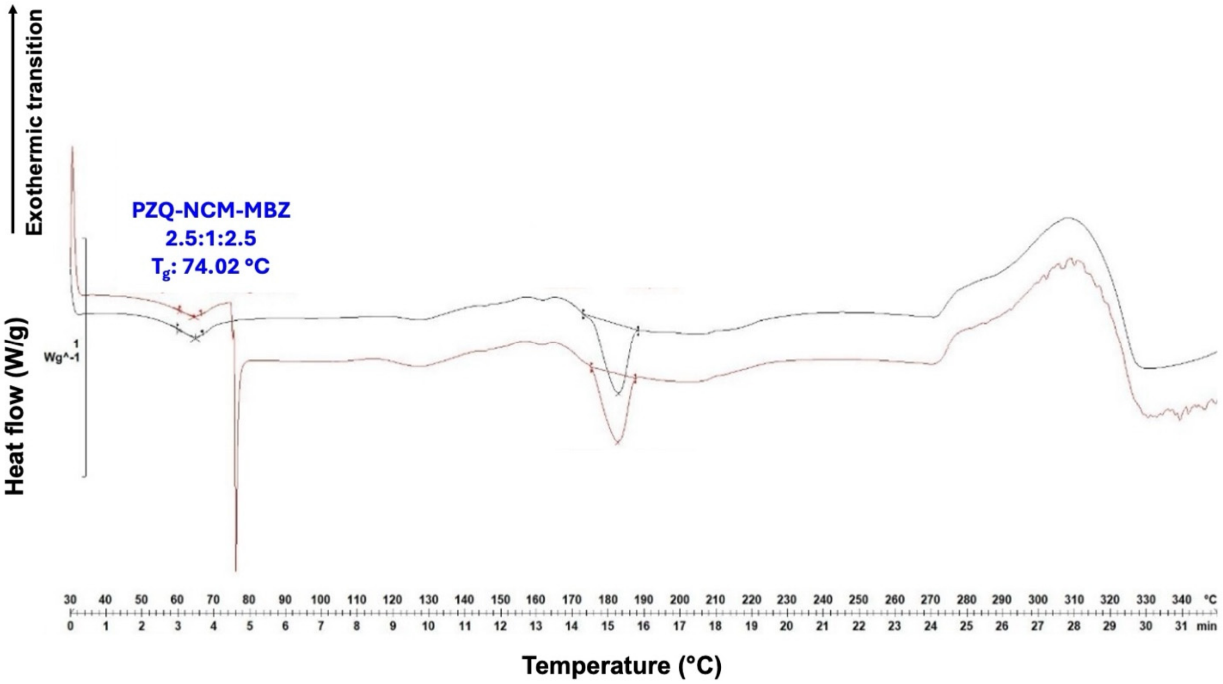

Figure S17. DSC curve showing T_g of exp. N° 5 (ternary PZQ-NCM-MBZ 2.5-1-2.5 system).

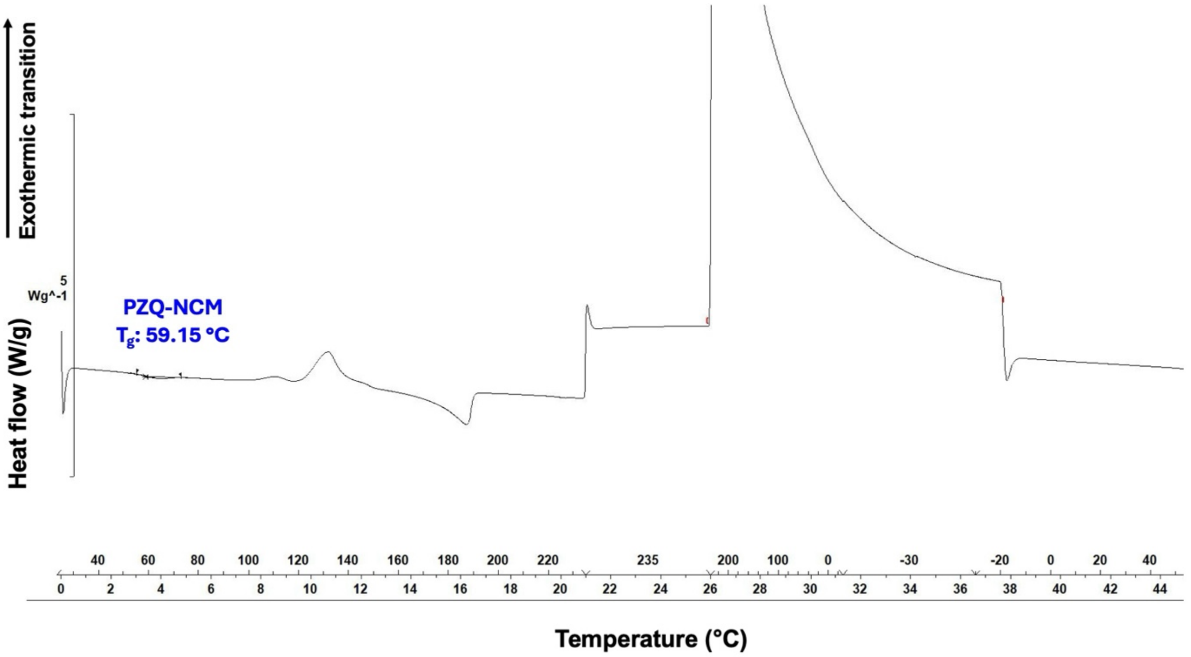

Figure S18. DSC curve showing T_g of exp. N° 6 (binary PZQ-NCM 1-1 system).

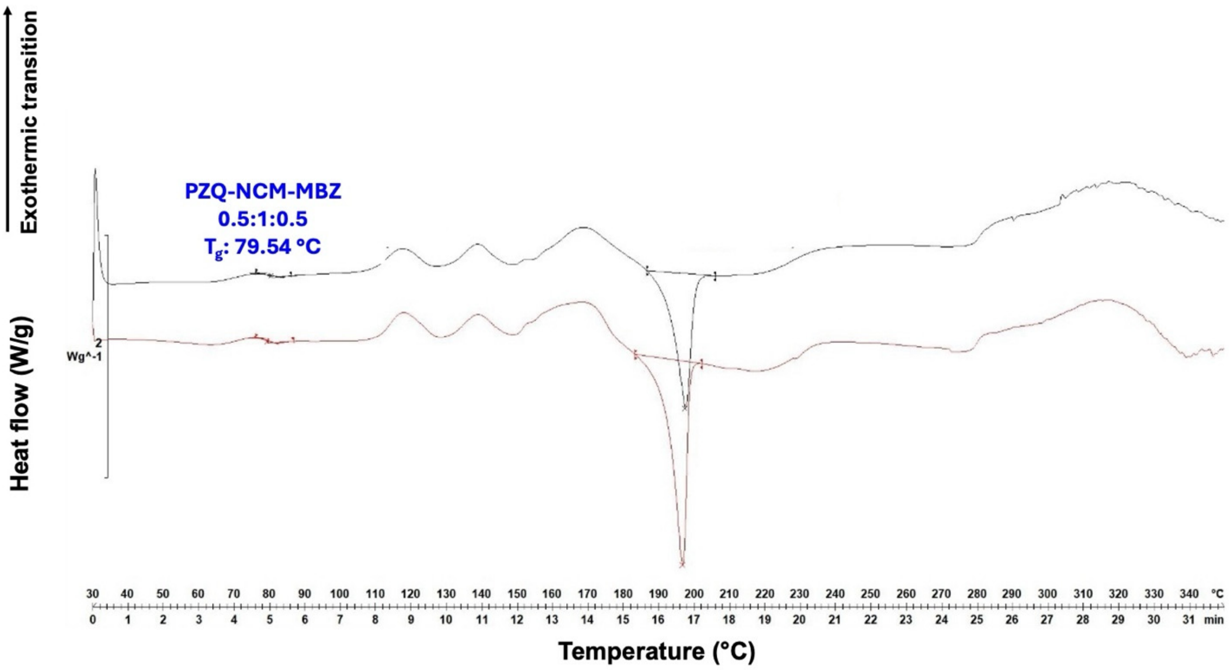

Figure S19. DSC curve showing T_g of exp. N° 7 (ternary PZQ-NCM-MBZ 0.5-1-0.5 system).

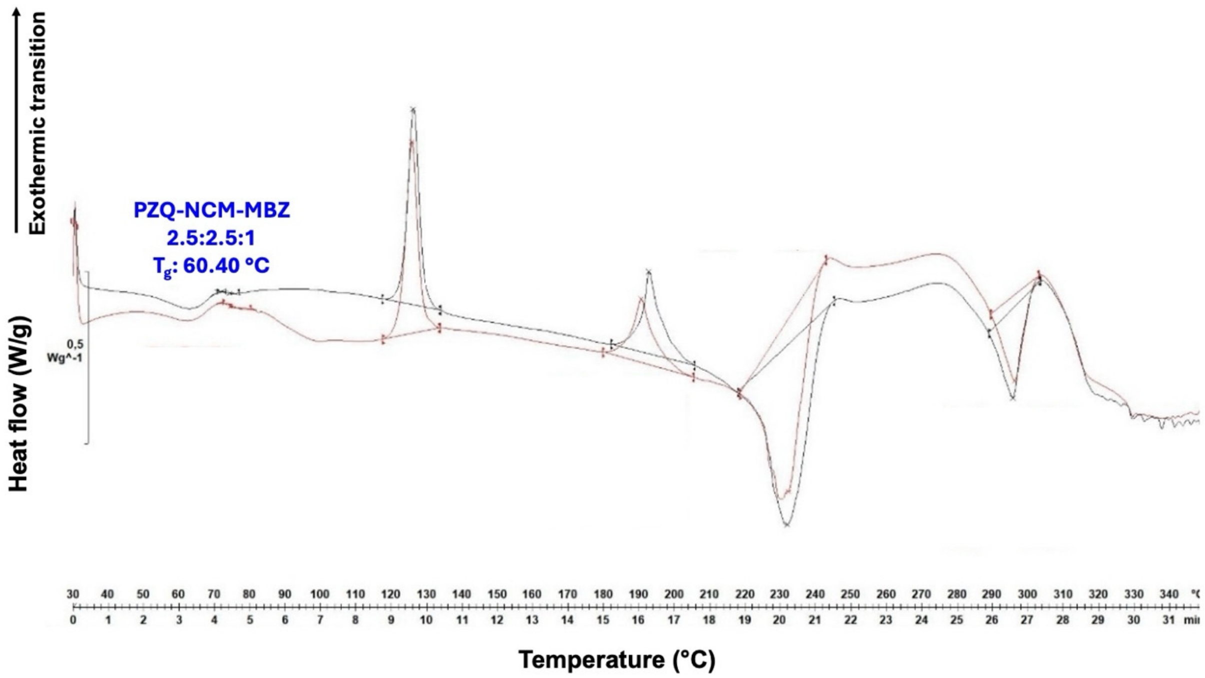

Figure S20. DSC curve showing T_g of exp. N° 8 (ternary PZQ-NCM-MBZ 2.5-2.5-1 system).

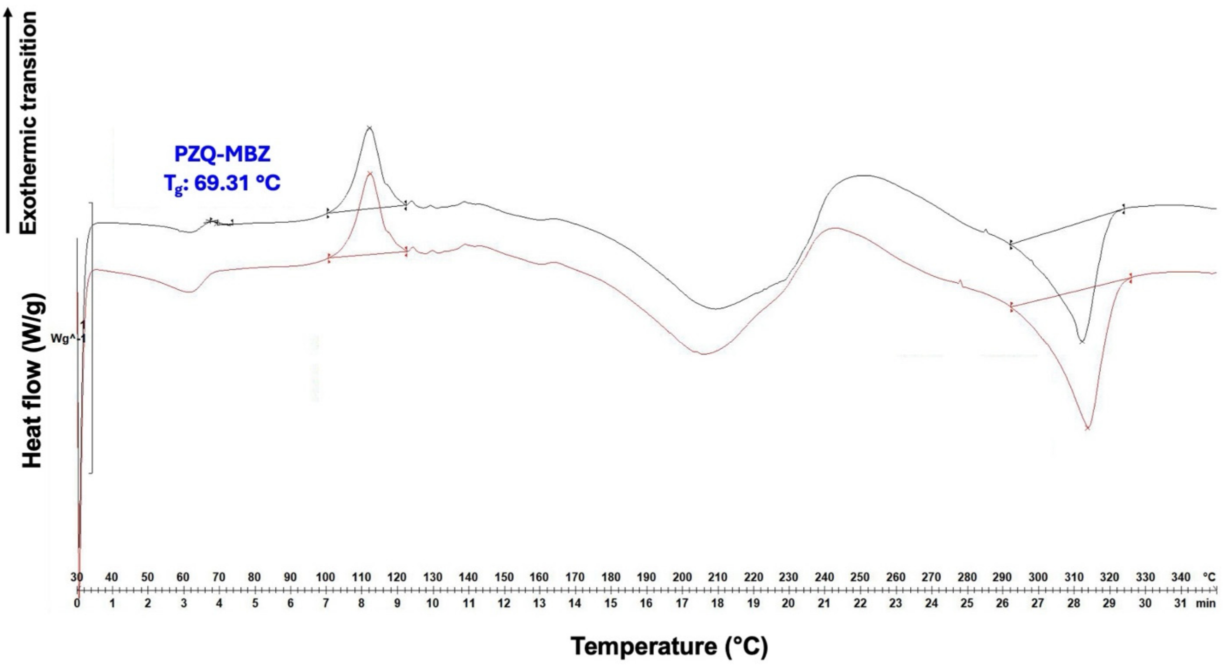

Figure S21. DSC curve showing T_g of exp. N° 9 (binary PZQ-MBZ 1-1 system).

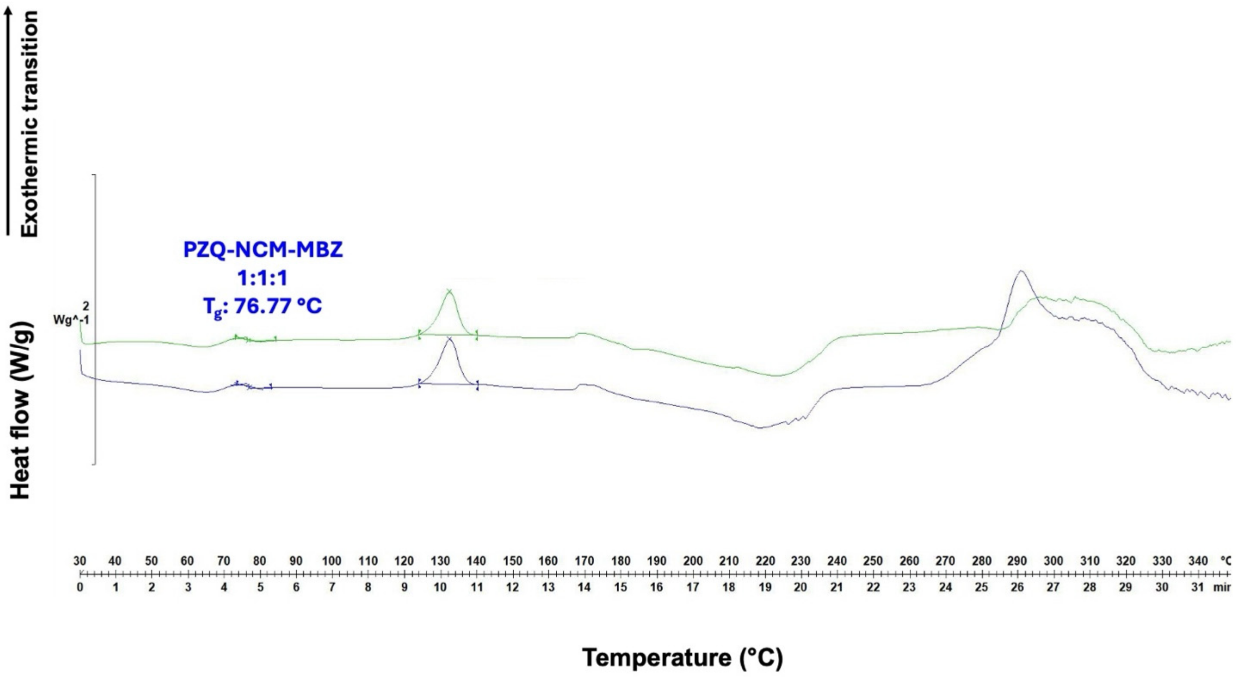

Figure S22. DSC curve showing T_g of exp. N° 10 (ternary PZQ-NCM-MBZ 1-1-1 system).

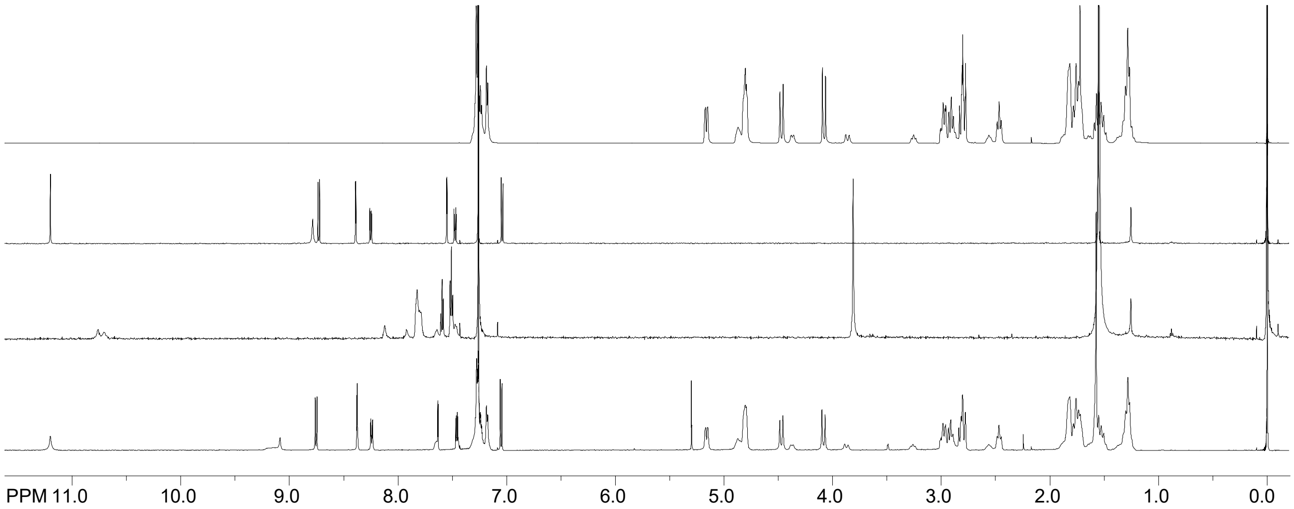

Figure S23. ^{1}H-NMR analysis (from top to bottom) of pure PZQ, NCM and MBZ compared to the sample of exp. N° 1.

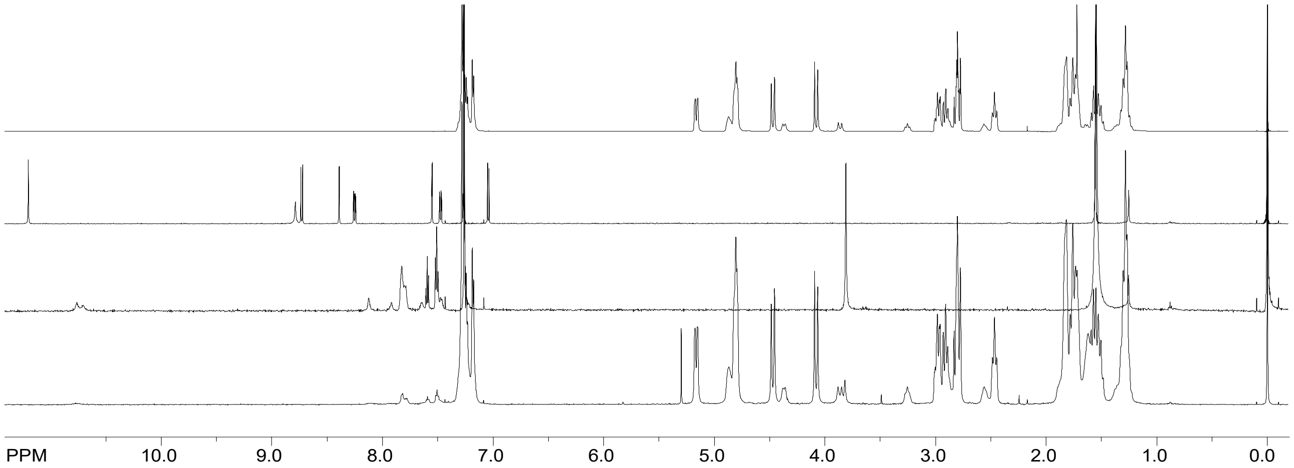

Figure S24. ^{1}H-NMR analysis (from top to bottom) of pure PZQ, NCM and MBZ compared to the sample of exp. N° 2.

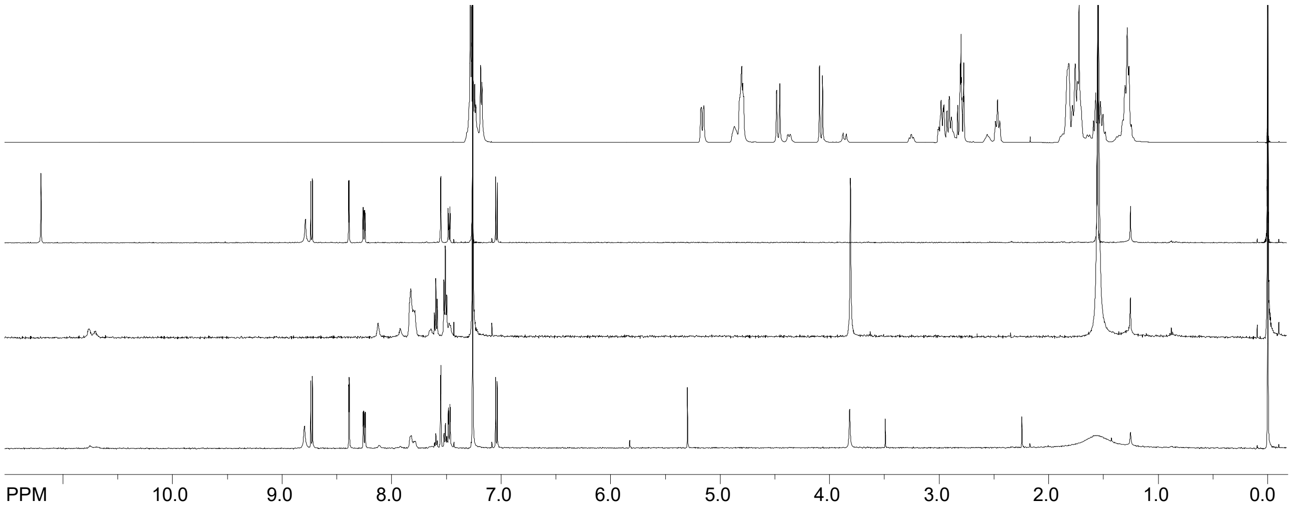

Figure S25. ^{1}H-NMR analysis (from top to bottom) of pure PZQ, NCM and MBZ compared to the sample of exp. N° 3.

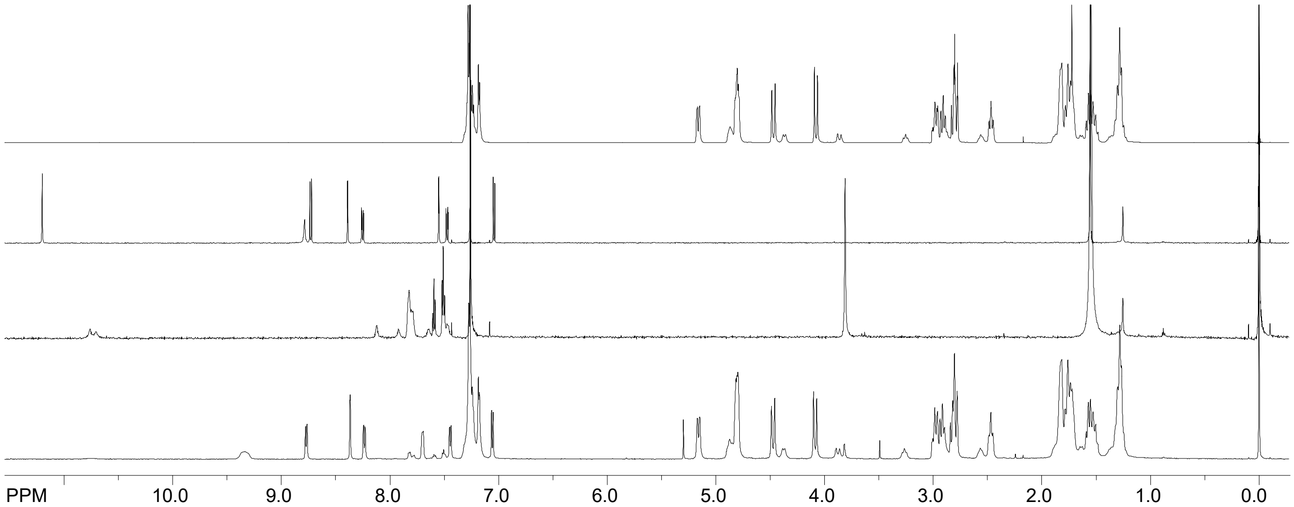

Figure S26. ^{1}H-NMR analysis (from top to bottom) of pure PZQ, NCM and MBZ compared to the sample of exp. N° 4.

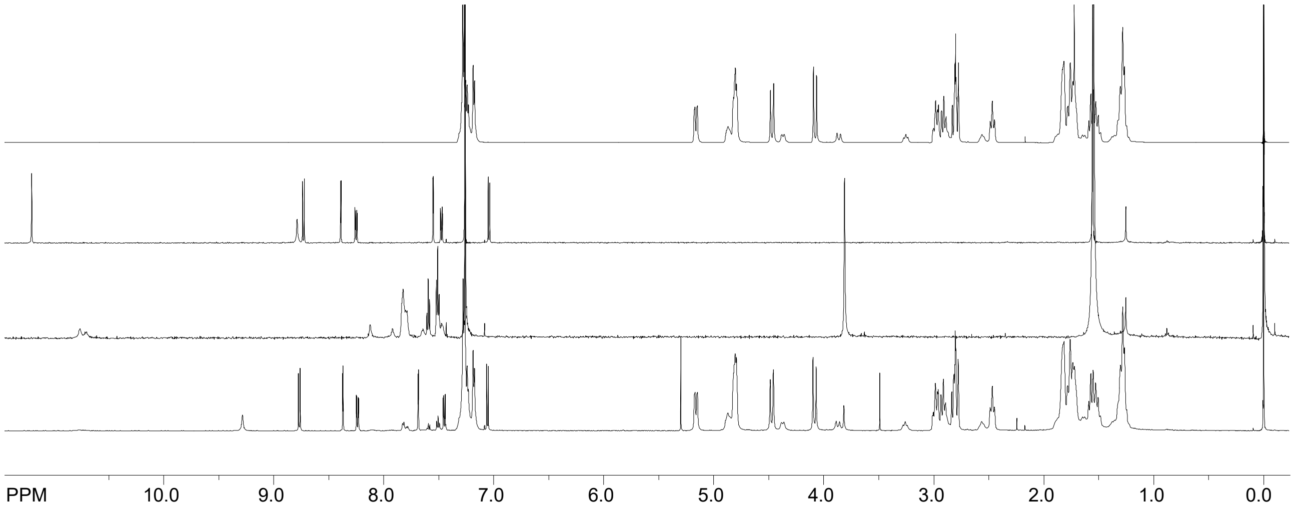

Figure S27. ^{1}H-NMR analysis (from top to bottom) of pure PZQ, NCM and MBZ compared to the sample of exp. N° 5.

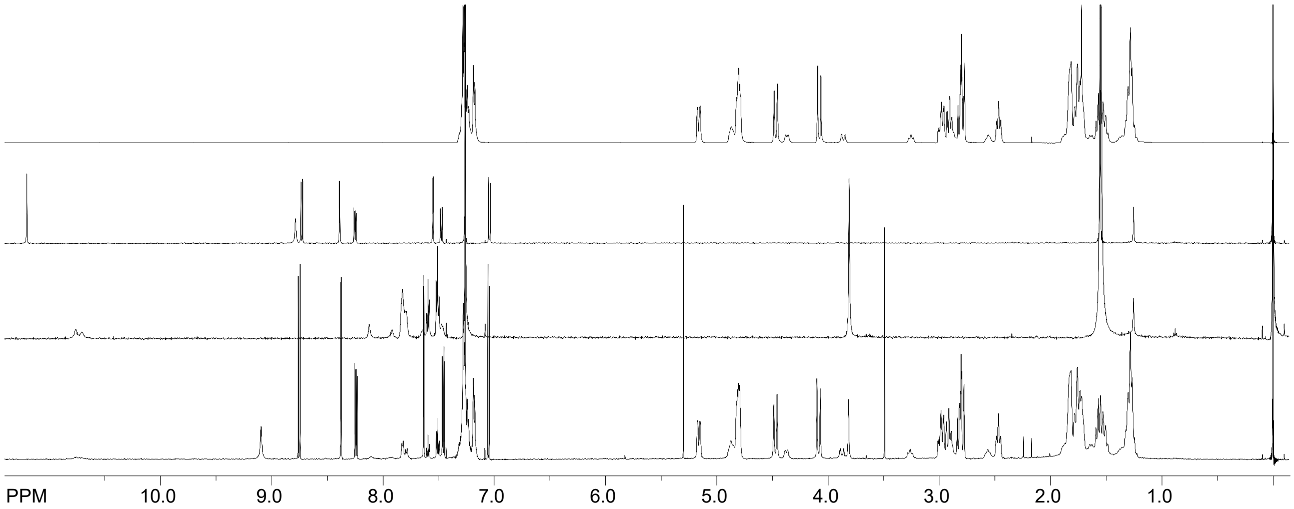

Figure S28. ^{1}H-NMR analysis (from top to bottom) of pure PZQ, NCM and MBZ compared to the sample of exp. N° 6.

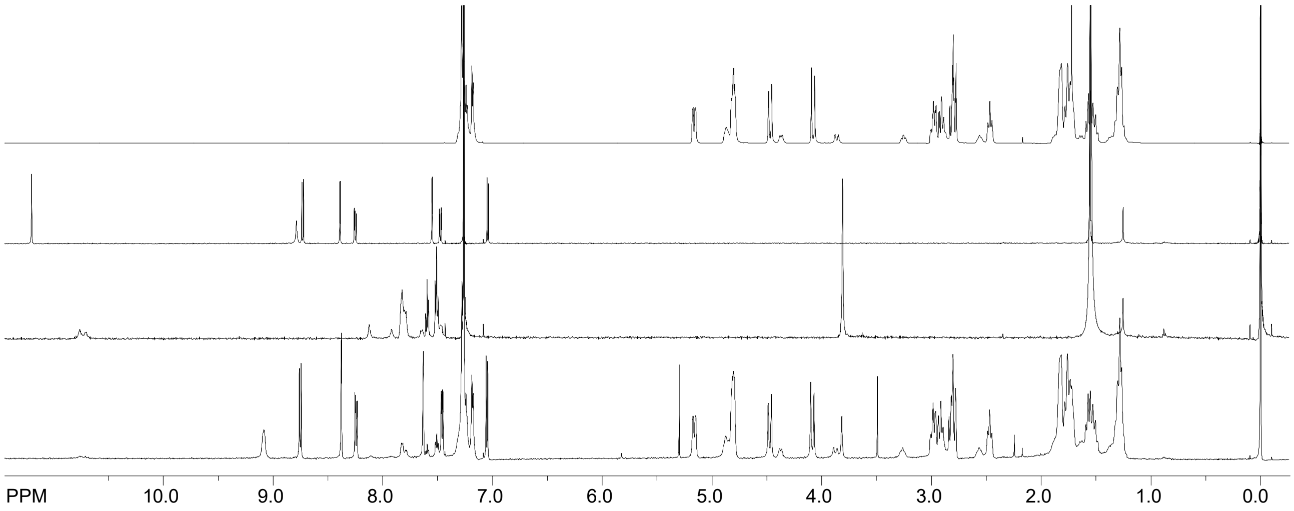

Figure S29. ^{1}H-NMR analysis (from top to bottom) of pure PZQ, NCM and MBZ compared to the sample of exp. N° 7.

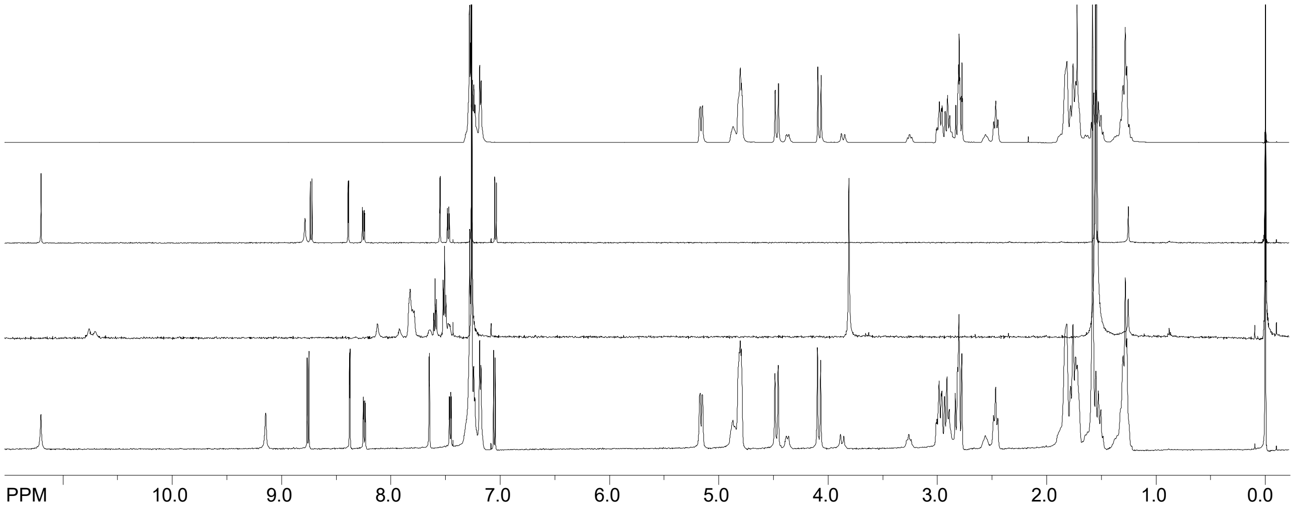

Figure S30. ^{1}H-NMR analysis (from top to bottom) of pure PZQ, NCM and MBZ compared to the sample of exp. N° 8.

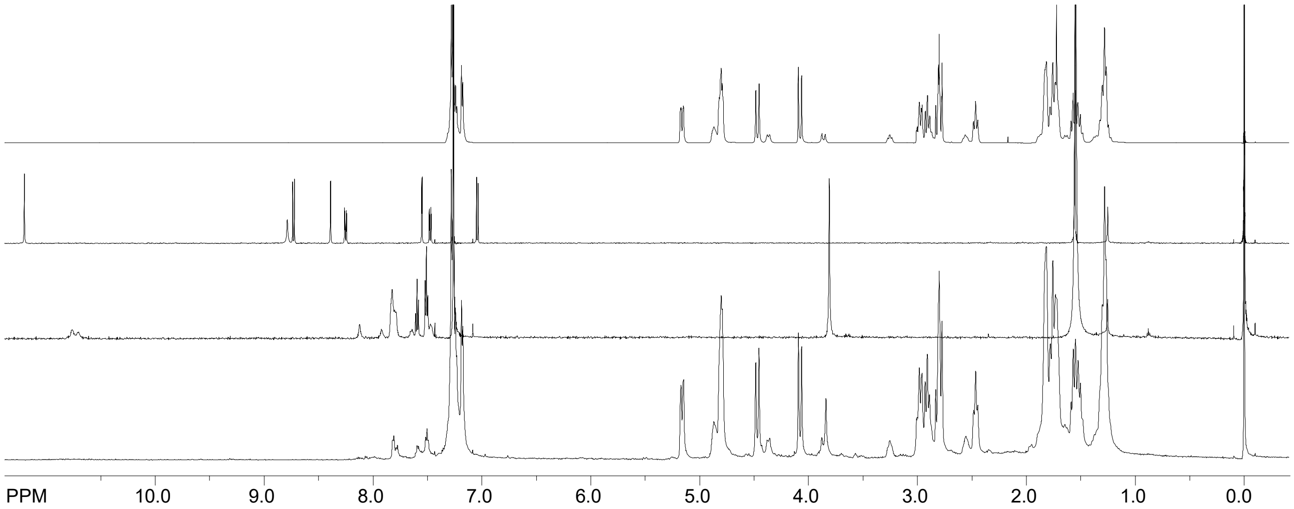

Figure S31. ^{1}H-NMR analysis (from top to bottom) of pure PZQ, NCM and MBZ compared to the sample of exp. N° 9.

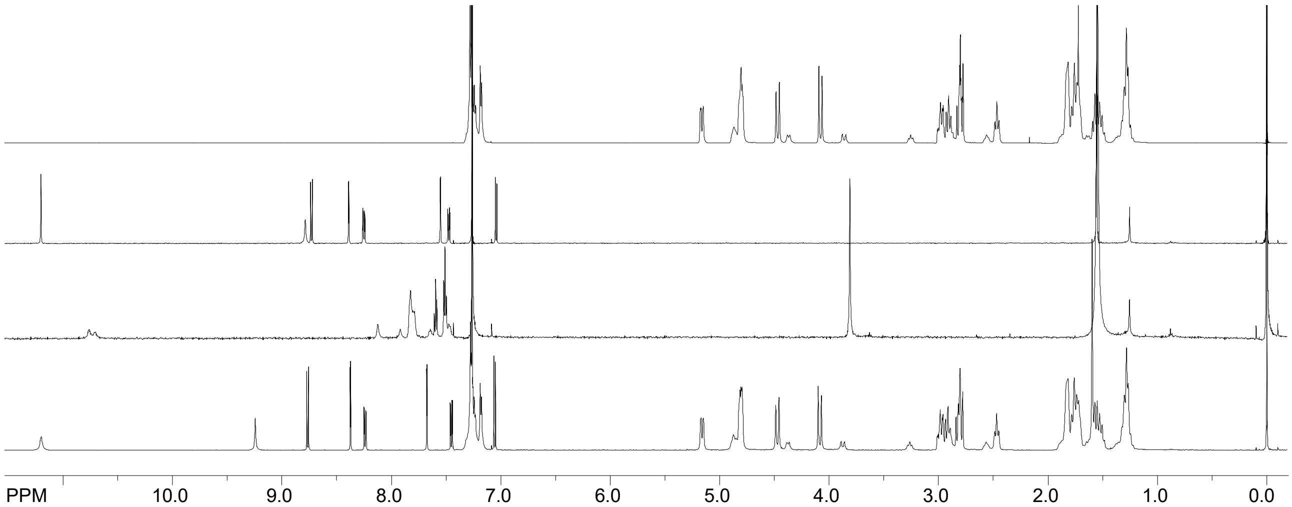

Figure S32. ^{1}H-NMR analysis (from top to bottom) of pure PZQ, NCM and MBZ compared to the sample of exp. N° 10.

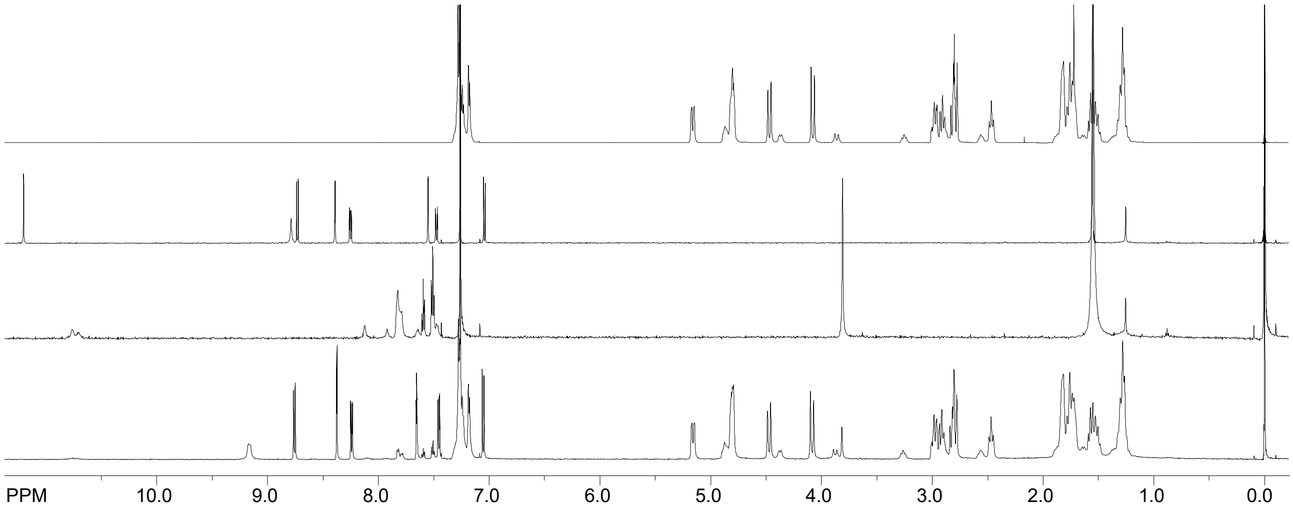

Figure S33. ^{1}H-NMR analysis (from top to bottom) of pure PZQ, NCM and MBZ compared to the sample of exp. N° 11.

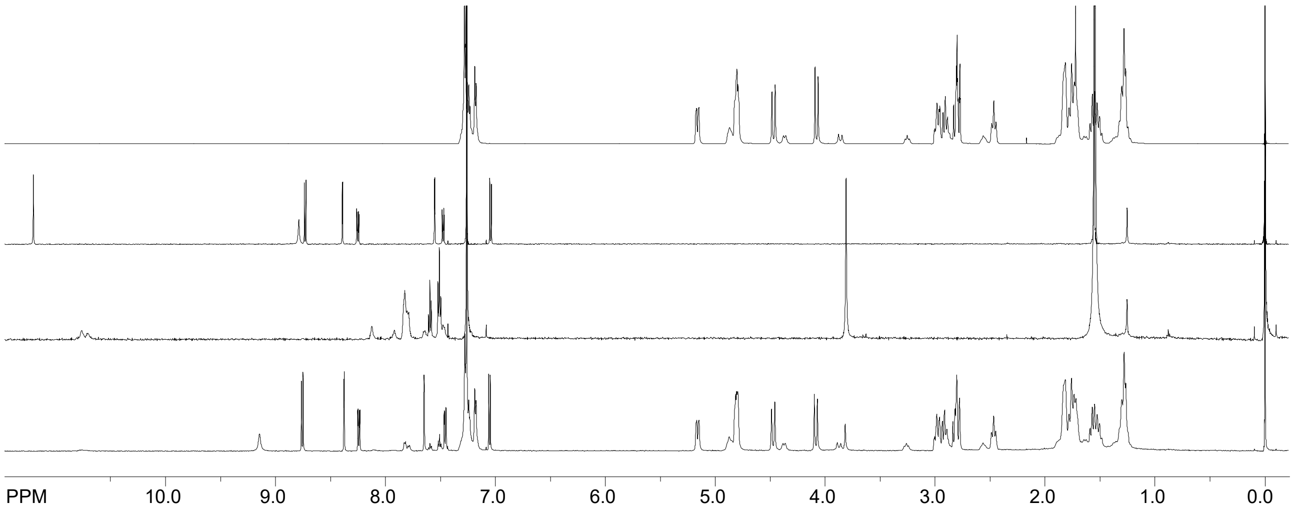

Figure S34. ^{1}H-NMR analysis (from top to bottom) of pure PZQ, NCM and MBZ compared to the sample of exp. N° 12.

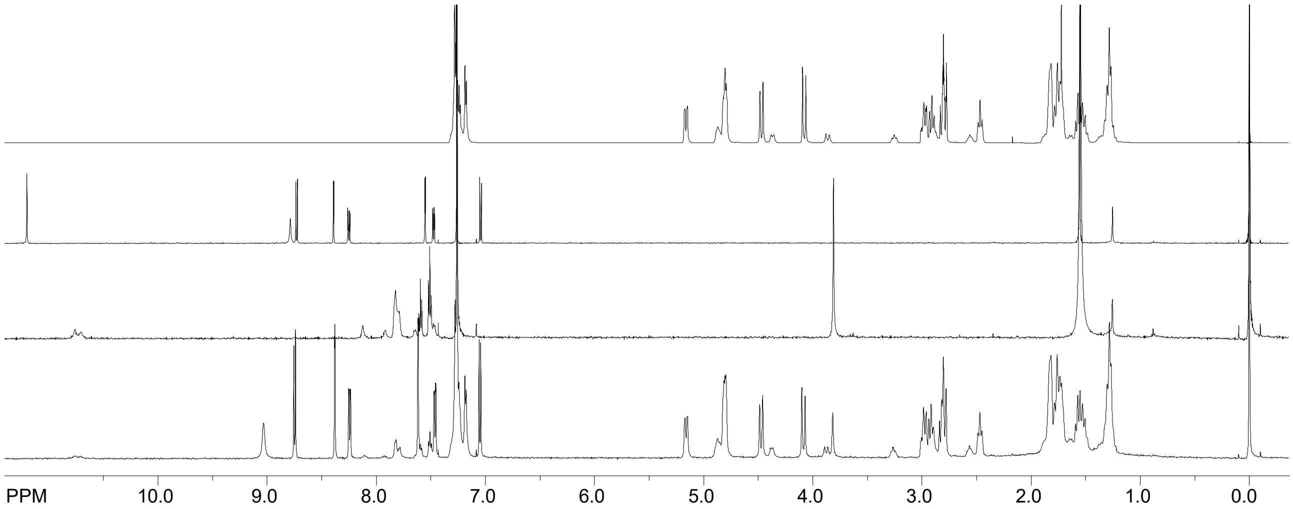

Figure S35. ^{1}H-NMR analysis (from top to bottom) of pure PZQ, NCM and MBZ compared to the sample of exp. N° 13.

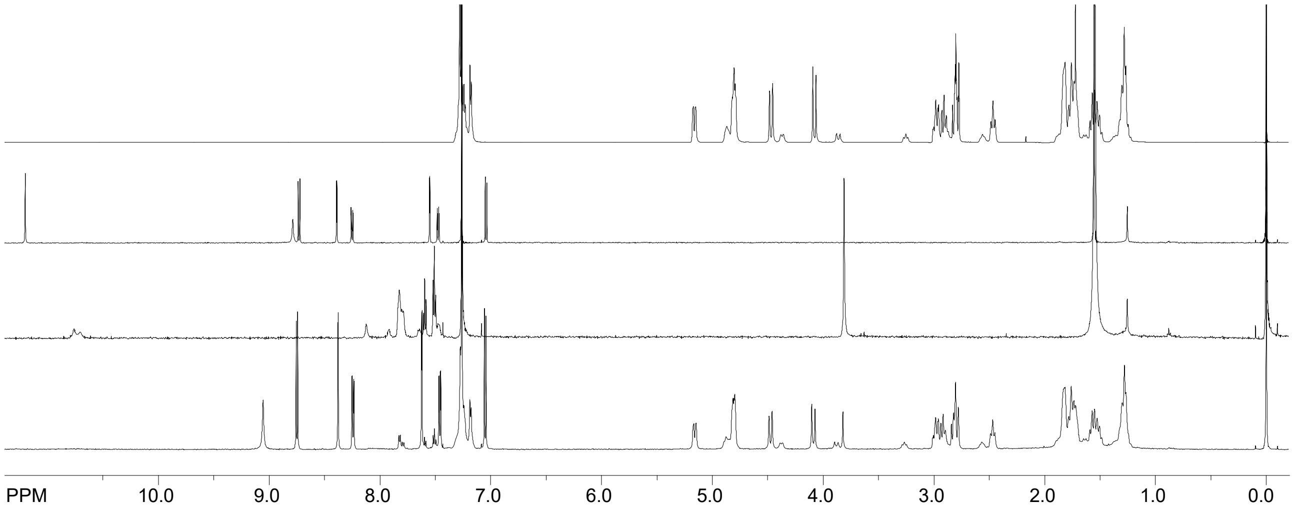

Figure S36. ^{1}H-NMR analysis (from top to bottom) of pure PZQ, NCM and MBZ compared to the sample of exp. N° 14.

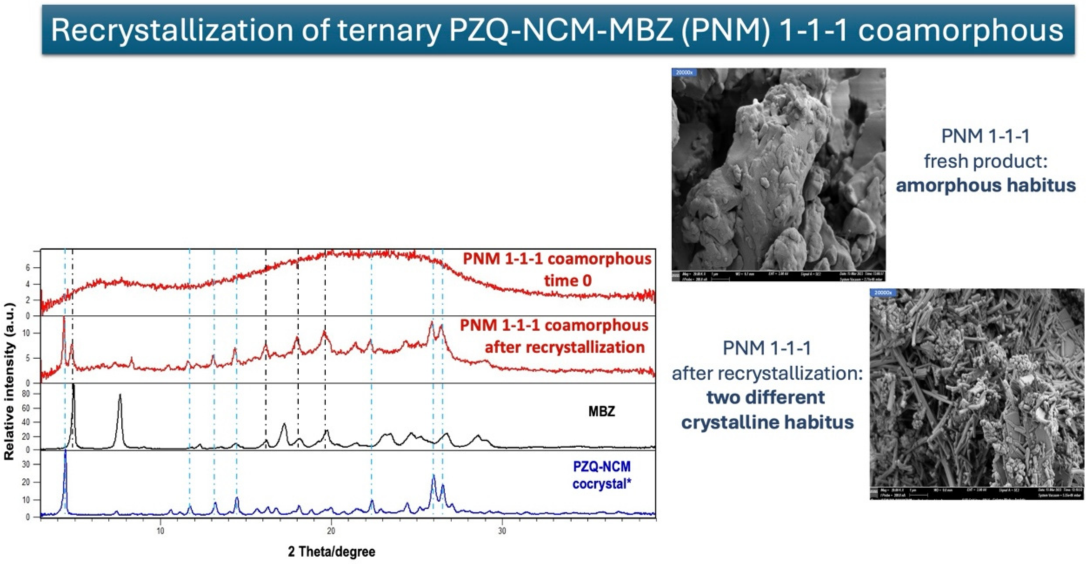

Figure S37. Cartoon depicting the recrystallization of ternary PZQ-MBZ-NCM 1-1-1 coamorphous into a mixture of MBZ and PZQ-NCM 1-3 anhydrous cocrystal through SEM and PXRD. Black dotted lines represent MBZ reflections, while light blue dotted lines PZQ-NCM 1-3 anhydrous cocrystal reflections.
